# Combination therapy for high-volume *versus* low-volume metastatic hormone-sensitive prostate cancer: A systematic review and network meta-analysis

**DOI:** 10.3389/fphar.2023.1148021

**Published:** 2023-04-20

**Authors:** Tengteng Jian, Yang Zhan, Ying Yu, Kai Yu, Rui Hu, Jixue Wang, Ji Lu

**Affiliations:** ^1^ Department of Urology, The First Hospital of Jilin University, Changchun, China; ^2^ School of Life Sciences, Jilin University, Changchun, China

**Keywords:** rezvilutamide, high-volume, low-volume, triplet therapy, doublet therapy, metastatic hormone-sensitive prostate cancer (mHSPC), network meta-analysis, adverse events

## Abstract

**Purpose:** To conduct a systematic review and network meta-analysis (NMA) to compare the efficacy of currently available combination therapies in patients with metastatic hormone-sensitive prostate cancer (mHSPC).

**Methods:** Qualified publications were searched in the PubMed, Embase, and Cochrane CENTRAL databases. Overall survival (OS) and radiographic progression-free survival (rPFS) were indirectly compared and assessed using NMA and the surface under the cumulative ranking curve, respectively. Adverse events (AEs) were also compared.

**Results:** Eighteen publications from 12 trials were analyzed in the NMA. In the overall population, triplet therapy was ranked first for OS (hazard ratio [HR]: 0.57, 95% credible interval [CrI]: 0.48–0.67) and rPFS (HR: 0.33, 95% CrI:0.26–0.41) compared with androgen deprivation therapy (ADT) with or without standard non-steroidal antiandrogen. In high-volume mHSPC, triplet therapy was also ranked first in OS (HR, 0.57; 95% CrI:0.44–0.75) and rPFS(HR, 0.29; 95% CrI: 0.23–0.37). Specifically, abiraterone triplet therapy was ranked first in OS (HR, 0.52; 95% CrI:0.38–0.72) and rPFS (HR, 0.28; 95% CrI:0.21–0.38) among all therapies. ADT plus rezvilutamide was ranked first among doublet therapies (OS: HR, 0.58; 95% CrI:0.44–0.77; rPFS: HR, 0.44; 95% CrI:0.33–0.58). In low-volume mHSPC, doublet and triplet therapies were ranked first in OS (HR:0.68, 95% CrI:0.58–0.80) and rPFS (HR:0.37, 95% CrI:0.25–0.55), respectively. ADT plus apalutamide was ranked first in OS among all therapies (HR:0.53, 95% CrI:0.35–0.79), whereas enzalutamide triplet therapy was ranked first in rPFS (HR:0.27, 95% CrI:0.15–0.51). ADT plus rezvilutamide showed a relatively lower incidence of AE among all therapies (OR:1.00, 95% CrI:0.31–3.15), and a lower risk of specific AEs among doublet therapies, particularly regarding seizure (OR, 0.29; 95% CrI:0.01–8.18) and fatigue (OR, 0.96; 95% CrI:0.63–1.46). Docetaxel-based doublet or triplet therapies significantly increased the risk of any AEs or grade ≥3 AEs.

**Conclusion:** Triplet therapy was the best treatment option for the overall population. In high-volume mHSPC, triplet therapy and ADT plus rezvilutamide had the greatest potential to benefit patients. Patients with low-volume mHSPC were most likely to benefit from ADT plus androgen receptor-targeted agents. Triplet therapy was associated with a higher risk of AEs than the other therapies.

**Systematic Review Registration:** https://www.crd.york.ac.uk/prospero/display_record.php?ID=CRD42022375347, identifier PROSPERO:CRD42022375347.

## 1 Introduction

Globally, prostate cancer (PCa) is the most frequently diagnosed solid tumor and the second leading cause of cancer-related deaths among men (Sung et al., 2021). The 5-year survival rate for patients with PCa at any stage was 98%. However, the 5-year survival rate for PCa patients with metastatic disease is approximately only 30% (Siegel et al., 2022).

As a standard treatment for metastatic hormone-sensitive prostate cancer (mHSPC), single androgen deprivation therapy agents including luteinizing hormone-releasing hormone agonists or antagonists have long been used (Kinsey et al., 2020). Since the introduction of other systemic agents such as docetaxel, abiraterone, enzalutamide, and apalutamide, the treatment landscape for mHSPC has changed dramatically, and the benefits have been demonstrated. In recent years, triplet therapies involving ADT plus docetaxel plus an androgen receptor-targeted agent (ARTA) have shown to be more effective (Fizazi et al., 2022; Smith et al., 2022). Besides, rezvilutamide, a novel ARTA, has been shown to improve the prognosis of patients with mHSPC with high-volume disease when combined with ADT (Gu et al., 2022). To compare these treatment options for mHSPC, several meta-analyses or network meta-analyses (NMAs) have been conducted (Jian et al., 2022; Maiorano et al., 2022; Mandel et al., 2022; Roy et al., 2022; Wenzel et al., 2022; Yanagisawa et al., 2022). However, rezvilutamide was rarely included in these studies, and few NMAs analyzed the efficacies of all current therapies based on the stratification of disease volume.

We therefore conducted a systematic review of all currently available therapies for mHSPC and compared the efficacy of different categories in the overall population. Subsequently, we indirectly compared the efficacy of specific therapies in patients with mHSPC with the high- and low-volume disease through NMA. Finally, the safety of all therapies was evaluated.

## 2 Materials and methods

### 2.1 Study design

The study protocol was registered in the International Prospective Register of Systematic Reviews (PROSPERO: CRD42022375347). In addition, this study followed the updated Preferred Reporting Items for Systematic Reviews and Meta-analyses reporting guidelines and its extension for NMA (Hutton et al., 2015; Page et al., 2021).

### 2.2 Literature search

We searched PubMed, Embase, and Cochrane CENTRAL databases to identify studies on ADT combination therapy for mHSPC published before November 2022. MeSH terms and free words related to prostate cancer, metastasis, treatment, and randomized trials were used in the analysis. A review of clinical trial registries and relevant abstracts presented at major conferences, including those of the American Society of Clinical Oncology and the European Society of Medical Oncology, was also conducted. The detailed database search strategy is presented in Supplementary Table S1.

### 2.3 Inclusion and exclusion criteria

The systematic review included trials if 1) patients in the group or subgroup received combination therapy containing ADT, and 2) the trials reported efficacy in terms of overall survival (OS) or progression-free survival (PFS). Trials were excluded if they were 1) on castration-resistant PCa, 2) including patients without metastatic disease, or 3) observational studies, reviews, cohorts, author responses, and case reports. A preliminary screening was conducted based on the titles and abstracts of the articles. Those reports that showed potential relevance were reviewed in the full text, and their significance was confirmed after data extraction. Discrepancies were resolved by consensus among all co-authors.

### 2.4 Data collection

The following information was independently extracted from the included articles by two researchers: year of publication, trial name, number of patients, characteristics of patients, inclusion criteria, therapies, the primary endpoint, duration of follow-up, and outcome definitions. Furthermore, hazard ratios (HR) and 95% credible intervals (CrIs) associated with the primary endpoints (OS and PFS) and adverse events (AEs) were also collected. All disagreements in data collection were subject to the consensus of the co-authors.

### 2.5 Quality evaluation

The risk of bias for each trial was assessed using the Cochrane Collaboration tool. This tool assesses selection bias (random sequence generation and allocation concealment), performance bias, detection bias, attrition bias, reporting bias, and bias from other sources (Higgins et al., 2011). The certainty or quality of evidence was assessed using the Grading of Recommendations Assessment, Development, and Evaluation tool (Atkins et al., 2004; Guyatt et al., 2011). Evidence quality is classified into four levels: high, moderate, low, and very low. The certainty of evidence began as high, which could be downrated to moderate, low, or very low according to five domains (risk of bias, inconsistency, imprecision, indirectness, and publication bias).

A valid NMA must meet three assumptions: homogeneity, consistency, and transitivity (Salanti, 2012). According to the homogeneity assumption, in order to make direct comparisons among available trials, each intervention group should be sufficiently homogeneous. Based on the consistency assumption, direct head-to-head and indirect comparisons should produce consistent effect estimates. A treatment network should contain a closed loop of interventions. Specifically, the transitivity assumption requires that the included trials should be clinically and methodologically sufficiently comparable.

### 2.6 Statistical analyses

Network plots were used to illustrate the connectivity of the treatment network for OS and radiographic PFS (rPFS). A Bayesian NMA was conducted for indirect comparisons for efficacies using fixed- and random-effect models. Trials with high heterogeneity were excluded to ensure that I2 values were <50%. Relative treatment effects were expressed as HRs and 95% CrIs (Woods et al., 2010; van Valkenhoef et al., 2012). A surface under the cumulative ranking curve (SUCRA) was used to estimate the treatment ranking probability for each outcome (Salanti et al., 2011). For AEs, arm-based analyses were performed to estimate the odd ratios (ORs) and 95% CrI from the available raw data presented in the selected publications (van Valkenhoef et al., 2012). An NMA using fixed-effects models with a frequentist approach was performed for direct and indirect treatment comparisons for AEs (Shim et al., 2019). All statistical analyses were performed using the R version 4.1.2 (R Foundation for Statistical Computing, Vienna, Austria). Statistical significance was set at *p* < 0.05.

## 3 Results

### 3.1 Study selection and characteristics

**FIGURE 1:**
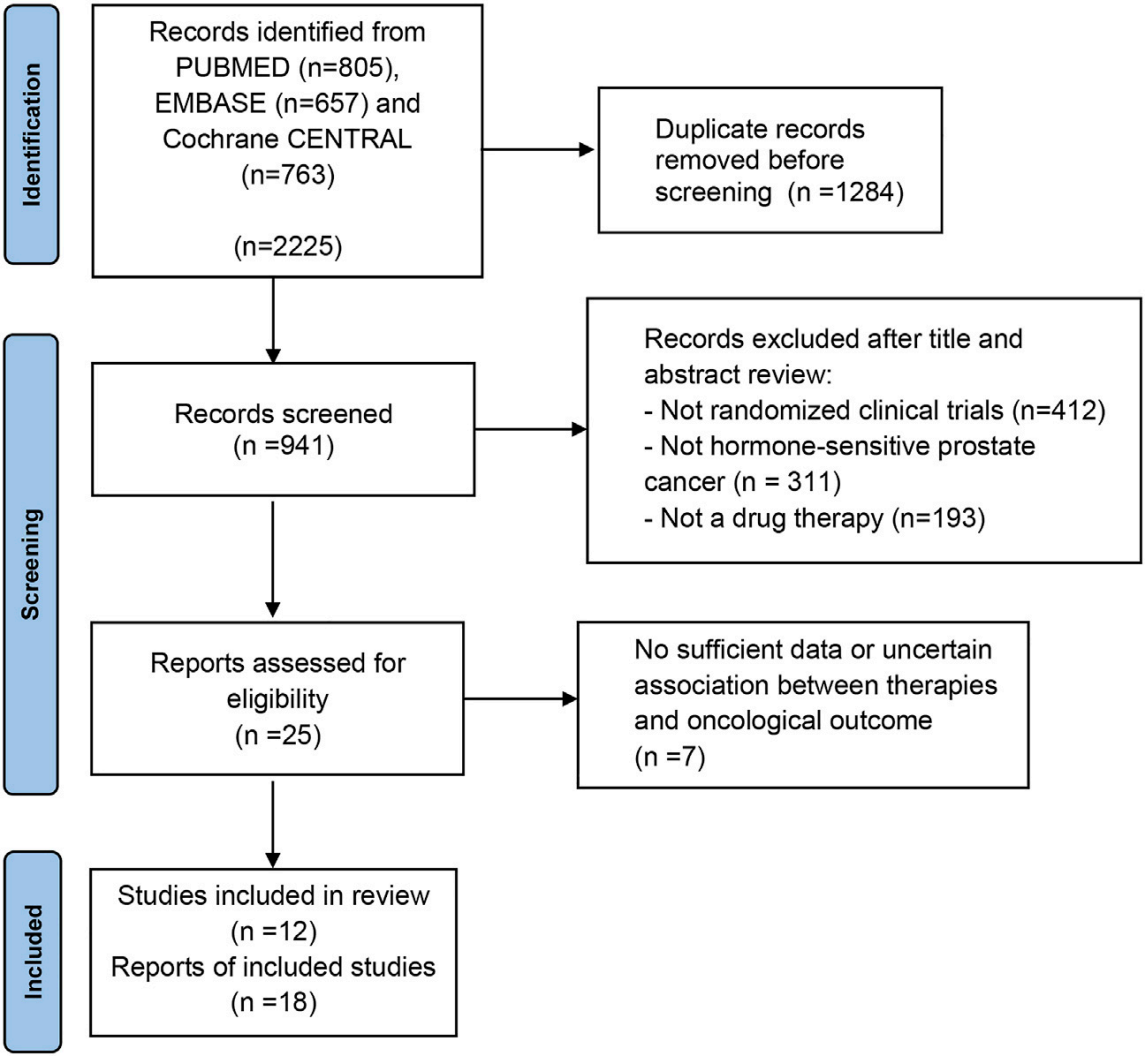
The PRISMA flow chart detailing the article selection process.

**FIGURE 2:**
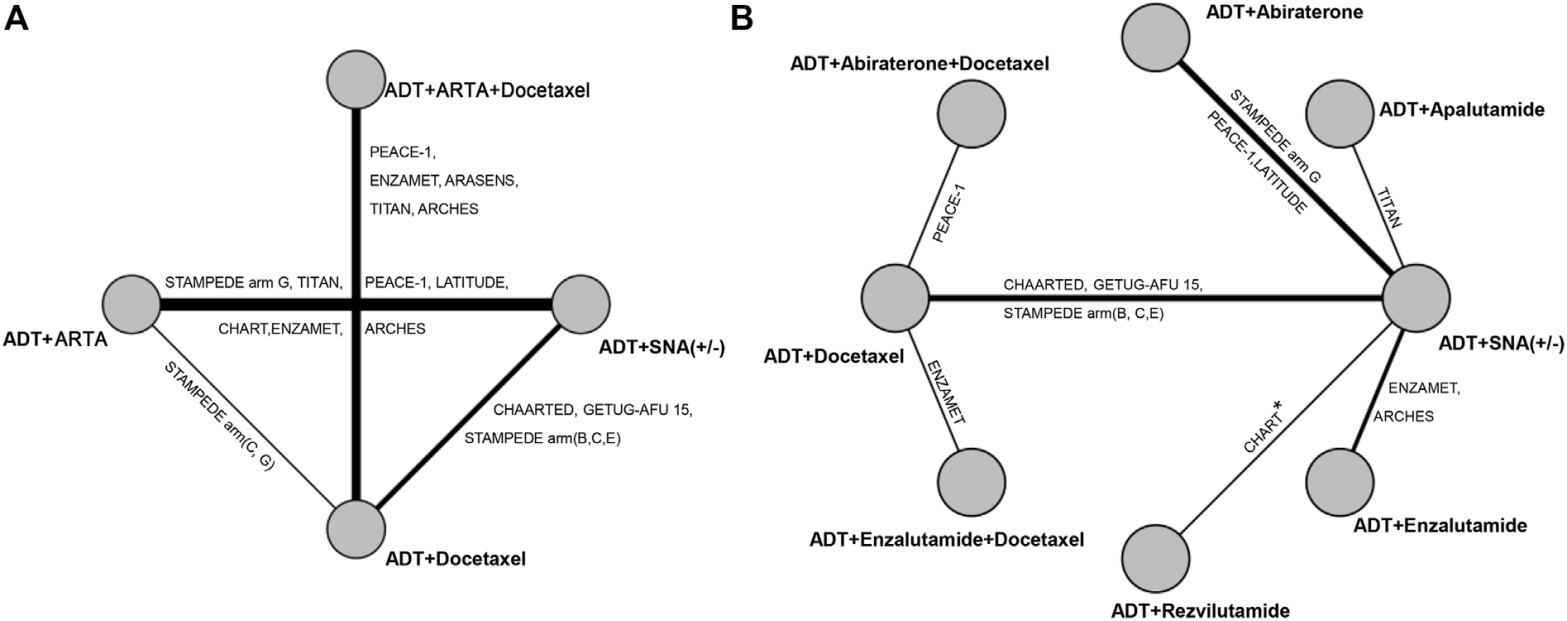
Network graph of trials comparison. **(A)** Comparison for categorized therapies. **(B)** Comparison for specific therapies in high- and low-volume disease. The nodes (circles) represent comparative therapy, and the edges (lines) show which therapies have been compared. The labels on the edges represent the names of RCTs comparing therapies. * Present at high-volume rather than at low-volume. RCTs, randomized controlled trials.

We identified 2,225 publications initially, and 941 publications were retained after removing duplicates. A total of 916 articles were excluded after title and abstract screening, and 25 were reviewed in the full text (Figure 1). Based on the selection criteria, 18 publications from 12 randomized controlled trials (RCTs) were identified (Gravis et al., 2013; Sweeney et al., 2015; Gravis et al., 2016; James et al., 2016; James et al., 2017; Kyriakopoulos et al., 2018; Sydes et al., 2018; Chi et al., 2019; Clarke et al., 2019; Davis et al., 2019; Fizazi et al., 2019; Hoyle et al., 2019; Chi et al., 2021; Armstrong et al., 2022; Azad et al., 2022; Fizazi et al., 2022; Gu et al., 2022; Smith et al., 2022). Table 1 summarizes the characteristics of the 12 studies included in the NMA. The studies were published between 2013 and 2022. Overall, 11,386 patients were included in these studies, and 10 therapies were evaluated, including ADT plus ARTA plus docetaxel (ADT plus abiraterone plus docetaxel, ADT plus darolutamide plus docetaxel, ADT plus enzalutamide plus docetaxel, and ADT plus apalutamide plus docetaxel), ADT plus ARTA (ADT plus abiraterone, ADT plus enzalutamide, ADT plus apalutamide, and ADT plus rezvilutamide), ADT plus docetaxel, and ADT with or without standard non-steroidal antiandrogen (SNA). Besides, 6,043 and 3,471 patients with high- and low-volume disease were included in the NMA, respectively. Figure 2 shows a network graph of the trial comparison.

**TABLE 1:** Characteristics of clinical trials included in the network meta-analyses.

Trials	Publications	Inclusion criteria	Overall population (C vs. E)	Control arm	Experimental arm	Percentage of high-volume (C vs. E)	Percentage of low-volume (C vs. E)	Median age, yr (range) (C vs. E)	Gleason score≥8 (C vs. E)	Performance status	Median follow-up (mo)	Primary endpoint
GETUG-AFU 15	Gravis et al. (2013), Gravis et al. (2016)	mHSPC No previous CTx	193/192	ADT	ADT + Docetaxel	47 vs. 48	53 vs. 52	64 (58–70) vs. 63 (57–68)	59% vs. 55%	ECOG 0–2	84	OS,rPFS
CHAARTED	Sweeney et al. (2015), Kyriakopoulos et al. (2018)	mHSPC No previous CTx	393/397	ADT	ADT + Docetaxel	64 vs. 66	36 vs. 34	62 (39–91) vs. 64 (36–88)	62% vs. 61%	ECOG 0–2	54	OS,cPFS
STAMPEDE arm (B,C,E)	James et al. (2016), Clarke et al. (2019)	mHSPC No previous CTx	724/362	ADT	ADT + Docetaxel	57 vs. 54	43 vs. 46	65(IQR:60–71)vs.65(IQR:60–70)	68% vs. 69%	WHO 0–2	78	OS, PFS
STAMPEDE arm G	James et al. (2017), Hoyle et al. (2019)	mHSPC or nodepositive PC or 2 risk factors or high-risk relapse	452/449	ADT	ADT + Abiraterone	51 vs. 54	49 vs. 46	67(IQR:63–72)vs.67(IQR:63–72)	74% vs. 75%	WHO 0–2	73	OS, PFS
STAMPEDE arm (C,G)	Sydes et al. (2018)	mHSPC No previous CTx	189/377	ADT + Docetaxel	ADT + Abiraterone	NA	NA	66(IQR:62–71)vs.66(IQR:61–70)	81% vs. 75%	WHO 0–2	48	OS, PFS
ENZAMET	Davis et al. (2019)	mHSPC	562/563	ADT + SNA	ADT + Enzalutamide	52 vs. 53	48 vs. 47	69 (64–75) vs. 69 (63–75)	57% vs. 60%	ECOG 0–2	34	OS, cPFS
		mHSPC	249/254	ADT + SNA + Docetaxel	ADT + Enzalutamide + Docetaxel	72 vs. 70	38 vs. 30	NA	NA	ECOG 0–2	34	OS, cPFS
LATITUDE	Fizazi et al. (2019)	High-risk mHSPC No previous CTx	597/602	ADT	ADT + Abiraterone	78 vs. 82	22 vs. 18	66.8 (±8.5) vs. 67.3 (±8.5)	97% vs. 98%	ECOG 0–2	52	OS, rPFS
TITAN	Chi et al. (2019), Chi et al. (2021)	mHSPC	527/525	ADT	ADT + Apalutamide	64 vs. 62	36 vs. 38	68 (43–90) vs. 69 (45–94)	68% vs. 67%	ECOG 0–1	44	OS, rPFS
ARCHES	Azad et al. (2022), Armstrong et al. (2022)	mHSPC	576/574	ADT	ADT + Enzalutamide	65 vs. 62	35 vs. 38	69 (45–94) vs. 68 (43–90)	65% vs. 67%	ECOG 0–1	45	OS,rPFS
PEACE-1	Fizazi et al. (2022)	mHSPC No previous CTx	589/583	ADT + RT (+/−)	ADT + Abiraterone + RT (+/−)	57 vs. 57	43 vs. 43	66 (59–72)vs. 67 (61–72)	77% vs. 75%	ECOG 0–2	53	OS, rPFS
		mHSPC No previous CTx	355/355	ADT + Docetaxel + RT (+/−)	ADT + Docetaxel + Abiraterone + RT (+/−)	65 vs. 63	35 vs. 37	66 (59–70)vs. 66 (60–70)	80% vs. 77%	ECOG 0–2	53	OS, rPFS
ARASENS	Smith et al. (2022)	mHSPC	655/651	ADT + Docetaxel	ADT + Darolutamide + docetaxel	NA	NA	67 (42–86)vs. 67 (41–89)	79% vs. 78%	ECOG 0–1	43	OS
CHART	Gu et al. (2022)	mHSPC No previous CTx	328/326	ADT + Bicalutamide	ADT + Rezvilutamide	100 vs. 100	NA	69 (64–75)vs. 69 (64–74)	78% vs. 85%	ECOG 0–1	29	OS, rPFS

Definitions and results of efficacy outcomes in NMA, are listed in Supplementary Tables S2, S3A.

Abbreviations: ADT, androgen-deprivation therapy; C, control arm; DOC, docetaxel; CTx, chemotherapy; ECOG PS, eastern cooperative oncology group performance status; E, experimental arm; NA, not available; OS, overall survival; rPFS, Radiographic progression-free survival; RT, radiotherapy; SNA, standard non-steroidal antiandrogen (bicalutamide, nilutamide or flutamide).

### 3.2 Quality evaluation

The risk of bias assessment results are shown in Supplementary Figure S1. All included studies were prospective RCTs. CHAARTED, CHART, ENZAMET, PEACE-1, GETUG-AFU 15, STAMPEDE arm (B, C, and E), STAMPEDE arm (C and G), and STAMPEDE arm G were open-label trials, and these studies are considered to have a potentially high risk of blindness to participants and investigators. Moreover, 4 of the 12 trials had an unclear risk of random sequence generation, and 8 of the 12 trials had an unclear risk of other biases. Detailed assessment criteria for each item in all included trials are presented in Supplementary Table S4. Considering the limitations of the study, the findings of the meta-analysis are moderate or low in evidential certainty. A detailed explanation of the level of evidence is provided in Supplementary Table S5.

Homogeneity analyses were conducted in the NMA when the same therapy was applied to multiple studies. It can be assessed using I^2^ statistics, with I^2^ < 25%, 25% ≤ I^2^ ≤ 50%, and I^2^ > 50% being interpreted as signifying low-, intermediate-, and high-level heterogeneity, respectively. Data from multiple trials was pooled without trials with high heterogeneity (Supplementary Figure S2). Consistency analysis showed that the direct and indirect comparisons were consistent for overall population among the therapies of ADT plus ARTA, ADT plus docetaxel, and ADT with or without SNA (Supplementary Figure S6). Due to the lack of a closed loop in specific therapies for patients with mHSPC with high- or low-volume disease, a comparison between direct and indirect evidence was not possible, and no local inconsistency analysis was required. Therefore, the assumption of consistency was met. In both clinical and methodological terms, the transferability assumption was met based on the inclusion and exclusion criteria and the data presented in Table 1.

### 3.3 Efficacy in the overall population

**FIGURE 3:**
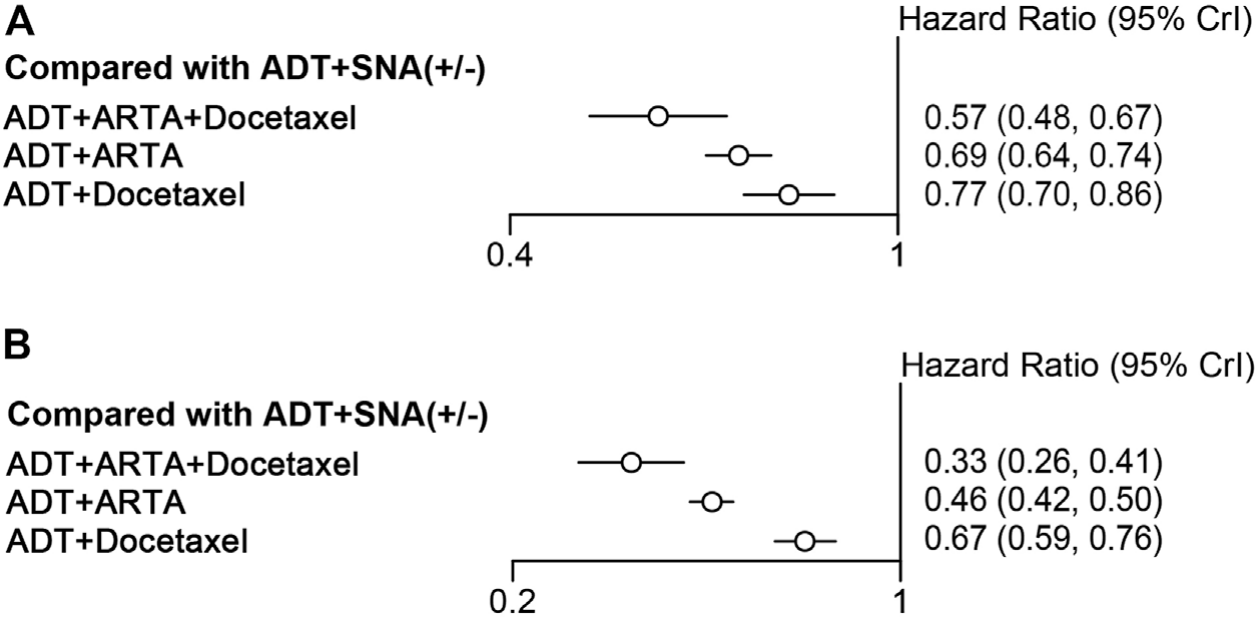
Comparison of categorized therapies for efficacies. **(A)** Forest plot representing HR for improving OS for combination therapy compared with ADT with or without SNA. **(B)** Forest plot representing HR for improving rPFS for combination therapy compared with ADT with or without SNA. OS, overall survival; ADT, androgen deprivation therapy; SNA, standard non-steroidal antiandrogen; rPFS, radiographic progression-free survival; HR, hazard ratio.

All 12 trials were included in the analysis. The efficacies of therapy were evaluated in different categories, including triplet therapy of ADT plus ARTA and docetaxel, ADT plus ARTA, and ADT plus docetaxel. Five trials involving triplet therapy were pooled, including ADT plus docetaxel plus enzalutamide, apalutamide, abiraterone, or darolutamide (ARASENS, ARCHES, ENZAMET, PEACE-1, and TITAN trials). For the rPFS analysis, only four trials were included since the rPFS data on darolutamide triplet therapy were not available in the ARASENS trial. Also, seven trials were pooled in OS and rPFS analyses of doublet therapy with ADT plus ARTA, including abiraterone, enzalutamide, apalutamide, and rezvilutamide (ARCHES, CHART, ENZAMET, LATITUDE, PEACE-1, STAMPEDE arm G, and TITAN trials). There were three trials involving ADT plus docetaxel (CHAARTED, GETUG-AFU 15, and STAMPEDE arm [B, C, and E] trials). Detailed information is provided in Supplementary Figure S2. In comparison with ADT with or without SNA, all combined therapies improved both OS and rPFS in the overall population. Triplet therapy was ranked first in both OS and rPFS improvements (HR: 0.57, 95% CrI: 0.48–0.67; HR: 0.33, 95% CrI:0.26–0.41), with a reduction in risks by 43% and 67% than ADT with or without SNA, respectively (Figure 3). Following triplet therapy, the doublet therapy of ADT plus ARTA and ADT plus docetaxel were ranked second and third, respectively, in terms of OS and rPFS.

### 3.4 Efficacy in patients with high-volume disease

**FIGURE 4:**
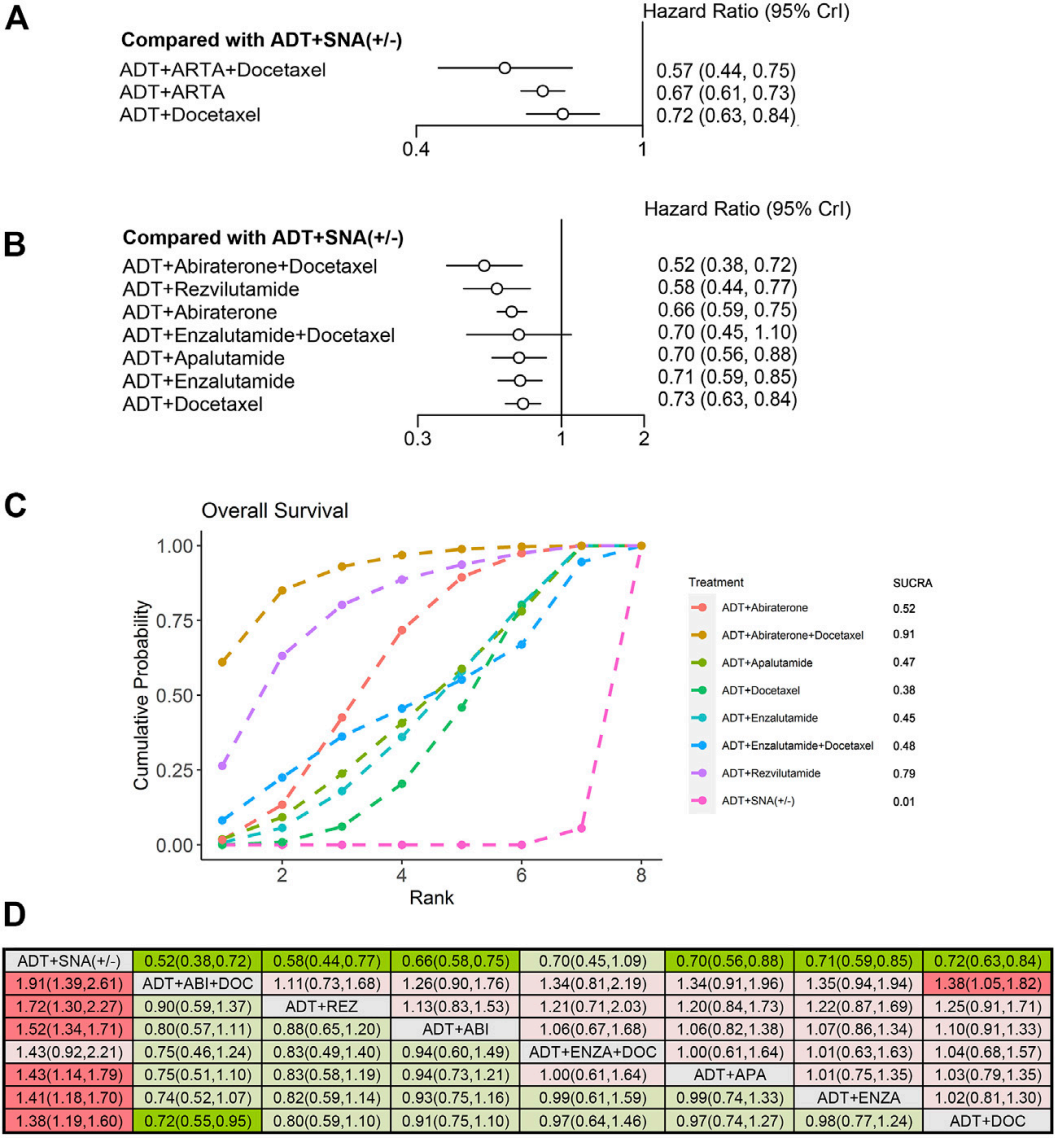
Comparison of therapies for improving OS in patients with high-volume disease. **(A)** Forest plot representing HR for combination therapy compared with ADT with or without SNA. **(B)** Forest plot representing HR for specific therapy compared with ADT with or without SNA. **(C)** SUCRA plot showing the treatment ranking of specific therapies. **(D)** League table of NMA comparing the OS. Comparison is located at the intersection of the column-defining treatment and the row-defining treatment. The results are presented in HR with 95% CrI. HR > 1 (red) favors row-defining treatment, and HR < 1 (green) favors column-defining treatment. The dark red or green represents the results with statistical significance. DOC, Docetaxel; ABI, Abiraterone, ENZA, Enzalutamide; APA, Apalutamide; REZ, Rezvilutamide; OS, overall survival; ADT, androgen deprivation therapy; SNA, standard non-steroidal antiandrogen; HR, hazard ratio; NMA, network meta-analysis; SUCRA, surface under the cumulative ranking curve.

**FIGURE 5:**
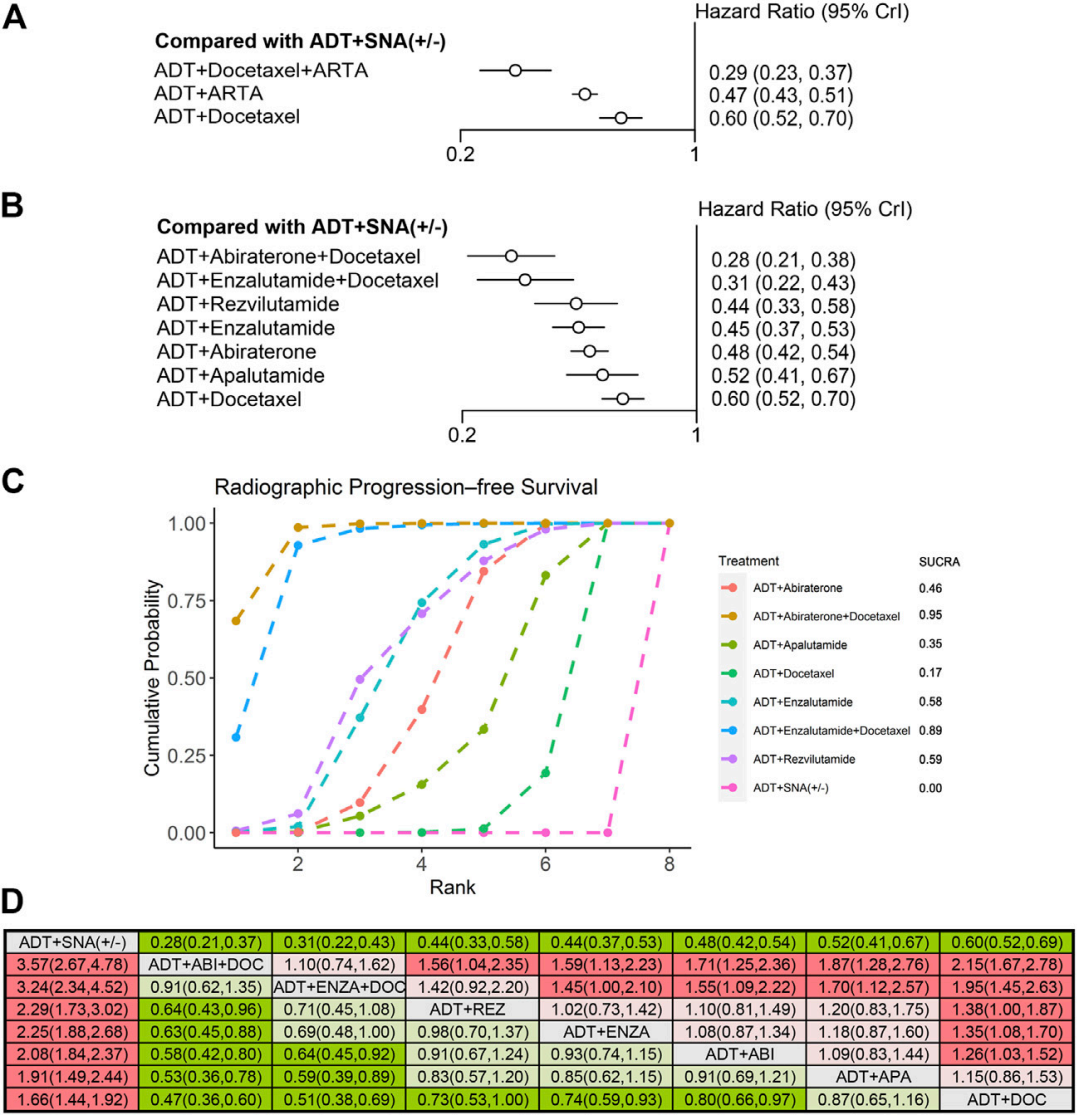
Comparison of therapies for improving rPFS in patients with high-volume disease. **(A)** Forest plot representing HR for combination therapy compared with ADT with or without SNA. **(B)** Forest plot representing HR for specific therapy compared with ADT with or without SNA. **(C)** SUCRA plot showing the treatment ranking of specific therapies. **(D)** League table of NMA comparing the rPFS. Comparison is located at the intersection of the column-defining treatment and the row-defining treatment. The results are presented in HR with 95% CrI. HR > 1 (red) favors row-defining treatment, and HR < 1 (green) favors column-defining treatment. The dark red or green represents the results with statistical significance. DOC, Docetaxel; ABI, Abiraterone, ENZA, Enzalutamide; APA, Apalutamide; REZ, Rezvilutamide; rPFS, radiographic progression-free survival; ADT, androgen deprivation therapy; SNA, standard non-steroidal antiandrogen; HR, hazard ratio; NMA, network meta-analysis; SUCRA, surface under the cumulative ranking curve.

Ten trials were qualified for analyzing the efficacy of therapy in mHSPC patients with high-volume disease. Overall, 6,043 patients with high-volume disease were included. First, the efficacy of triplet therapy with ADT plus ARTA plus docetaxel and doublet therapy with ADT plus ARTA or docetaxel in the high-volume disease subgroup were evaluated. Based on the trial design and available data, only two trials of triplet therapies involving enzalutamide and abiraterone were included (the ENZAMET and PEACE-1 trials). Seven trials of ADT plus ARTA and three with ADT plus docetaxel were included in this subgroup analysis, which was the same as overall analysis. Detailed information is provided in Supplementary Figure S3. As compared with ADT with or without SNA, all combined therapies improved OS and rPFS in patients with high-volume disease. The triplet therapy also showed the best efficacy on OS and rPFS (HR: 0.57, 95% CrI: 0.44–0.75; HR: 0.29, 95% CrI: 0.23–0.37), reducing risks by 43% and 71%, respectively (Figures 4A, 5A). The combination of ADT plus ARTA and ADT plus docetaxel was ranked second and third, respectively, as the same sequence as for the overall population.

To further compare the efficacy of different combinations of therapies in high-volume disease, we used NMA to assess the OS and rPFS improvement. Compared with ADT with or without SNA, six therapies showed improvements in OS, except for the triplet therapy of ADT plus enzalutamide plus docetaxel (Figure 4B). The triplet therapy of ADT plus abiraterone plus docetaxel showed the most significant improvements in OS (HR: 0.52, 95% CrI: 0.38–0.72), followed by the doublet therapy of ADT plus rezvilutamide (HR: 0.58, 95% CrI: 0.44–0.77), with a reduction in the risks of death by 48% and 42%, respectively. In the treatment ranking analysis, the combination of ADT with abiraterone and docetaxel had the highest probability of providing maximum OS, with a SUCRA of 0.91, followed by ADT plus rezvilutamide, with a SUCRA of 0.79 (Figure 4C). According to the pairwise comparisons in the OS, only triplet ADT plus abiraterone plus docetaxel was significantly superior to ADT plus docetaxel, and no other significant difference was observed between treatments (Figure 4D).

Compared with ADT with or without SNA, all seven therapies improved rPFS (Figure 5B). The triplet therapy with ADT plus docetaxel plus abiraterone showed the most significant improvements in rPFS (HR: 0.28, 95% CrI: 0.21–0.38), followed by ADT plus docetaxel plus enzalutamide (HR: 0.31, 95% CrI: 0.22–0.43), and ADT plus rezvilutamide (HR: 0.44, 95% CrI:0.33–0.58), reducing risks by 72%, 69%, and 56%, respectively. According to the treatment ranking analysis, abiraterone triplet therapy had the highest probability of providing maximum rPFS (SUCRA, 0.95), followed by enzalutamide triplet therapy (SUCRA, 0.89), rezvilutamide doublet therapy (SUCRA, 0.59) and enzalutamide doublet therapy (SUCRA, 0.58) (Figure 5C). In the pairwise comparisons, although ADT plus docetaxel was better than ADT with or without SNA, it was worse than most triplet and doublet therapies and was not statistically different from ADT plus apalutamide. The results were consistent with previous analyses, showing that triplet therapies were more effective than most doublet therapies in patients with rPFS. There was no significant difference in rPFS improvement between any two doublet therapies of ADT plus ARTA (Figure 5D).

### 3.5 Efficacy in patients with low-volume disease

**FIGURE 6:**
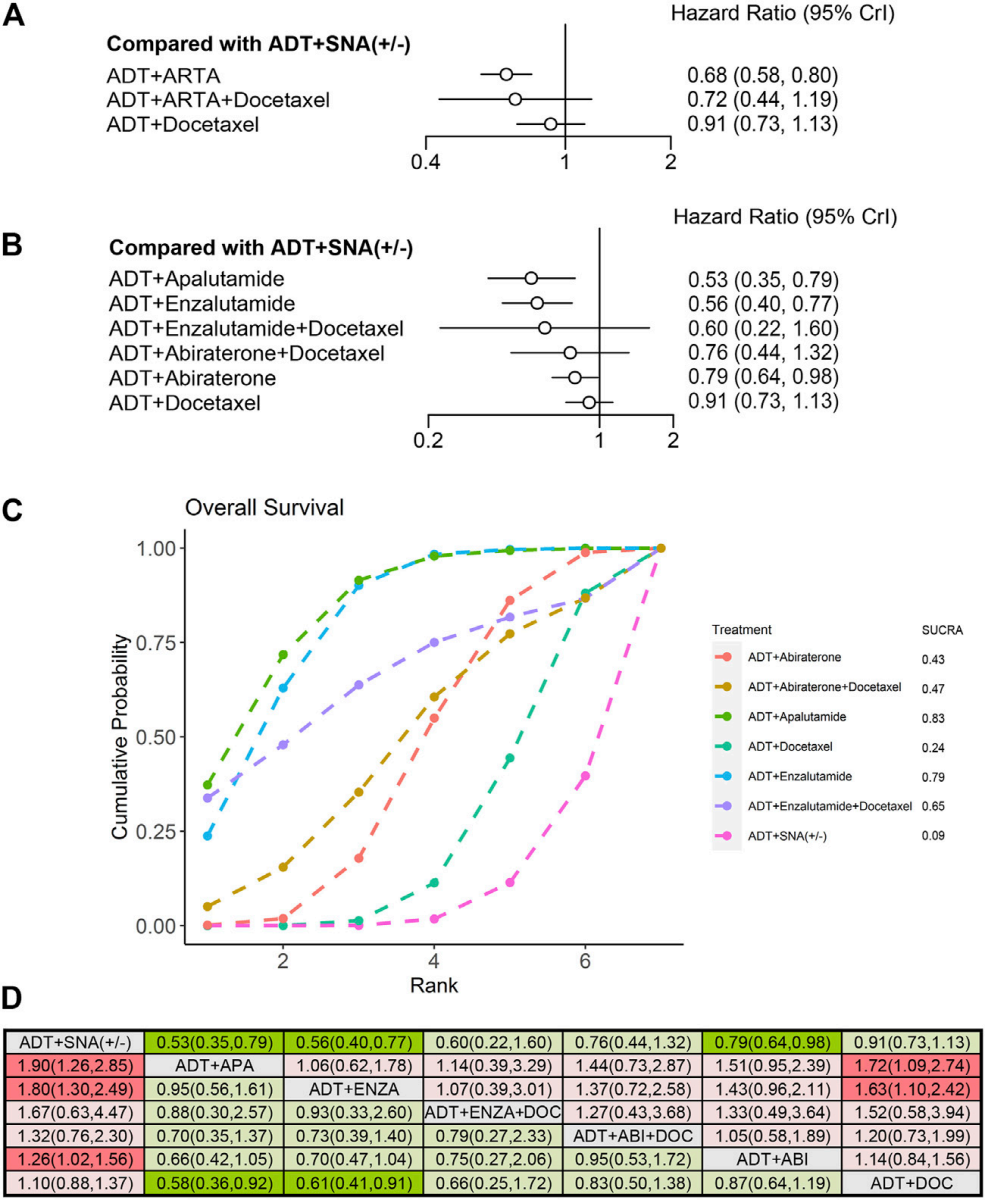
Comparison of therapies for improving OS in patients with low-volume disease. **(A)** Forest plot representing HR for combination therapy compared with ADT with or without SNA. **(B)** Forest plot representing HR for specific therapy compared with ADT with or without SNA. **(C)** SUCRA plot showing the treatment ranking of specific therapies. **(D)** League table of NMA comparing the OS. Comparison is located at the intersection of the column-defining treatment and the row-defining treatment. The results are presented in HR with 95% CrI. HR > 1 (red) favors row-defining treatment, and HR < 1 (green) favors column-defining treatment. The dark red or green represents the results with statistical significance. DOC, Docetaxel; ABI, Abiraterone, ENZA, Enzalutamide; APA, Apalutamide; OS, overall survival; ADT, androgen deprivation therapy; SNA, standard non-steroidal antiandrogen; HR, hazard ratio; NMA, network meta-analysis; SUCRA, surface under the cumulative ranking curve.

**FIGURE 7:**
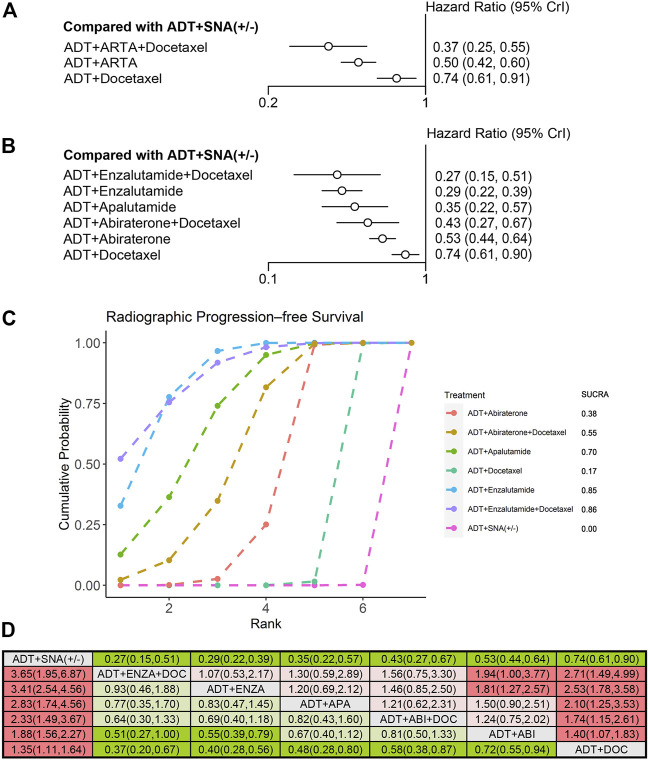
Comparison of therapies for improving rPFS in patients with low-volume disease. **(A)** Forest plot representing HR for combination therapy compared with ADT with or without SNA. **(B)** Forest plot representing HR for specific therapy compared with ADT with or without SNA. **(C)** SUCRA plot showing the treatment ranking of specific therapies. **(D)** League table of NMA comparing the rPFS. Comparison is located at the intersection of the column-defining treatment and the row-defining treatment. The results are presented in HR with 95% CrI. HR > 1 (red) favors row-defining treatment, and HR < 1 (green) favors column-defining treatment. The dark red or green represents the results with statistical significance. DOC, Docetaxel; ABI, Abiraterone, ENZA, Enzalutamide; APA, Apalutamide; rPFS, radiographic progression-free survival; ADT, androgen deprivation therapy; SNA, standard non-steroidal antiandrogen; HR, hazard ratio; NMA, network meta-analysis; SUCRA, surface under the cumulative ranking curve.

Nine trials qualified for analyzing the efficacy of mHSPC in patients with low-volume disease. Overall, 3,471 patients with low-volume disease were included. The efficacies of triplet therapy with ADT plus ARTA plus docetaxel and doublet therapy with ADT plus ARTA or docetaxel in the subgroup of low-volume disease were evaluated. OS and rPFS improvements were analyzed from the ENZAMET and PEACE-1 trials with enzalutamide and abiraterone triplet therapies. In addition, six trials with doublet therapy of ADT plus ARTA were pooled into the OS analysis, while only four trials were pooled into the rPFS analysis due to high heterogeneity in the ARCHES and ENZAMET trials. Three trials involving ADT plus docetaxel were included. Detailed information is provided in Supplementary Figure S4. Contrary to high-volume disease, although all three combination therapies improved rPFS in low-volume disease, only ADT plus ARTA improved OS over ADT with or without SNA. ADT plus ARTA significantly prolonged both OS (HR: 0.68, 95% CrI: 0.58–0.80) and rPFS (HR: 0.50, 95% CrI: 0.42–0.60), with a reduction in risks by 32% and 50%, respectively (Figures 6A, 7A).

An NMA was conducted to evaluate the OS and rPFS improvement of specific therapies for low-volume disease. Compared with ADT with or without SNA, the doublet therapy of ADT plus apalutamide (HR: 0.53, 95% CrI: 0.35–0.79) and ADT plus enzalutamide (HR: 0.56, 95% CrI: 0.40–0.77) demonstrated the best improvement in OS (Figure 6B). In the treatment ranking analysis, both of them had the highest probability of providing maximum OS, with SUCRAs of 0.83 and 0.79, respectively (Figure 6C). However, no significant difference was observed in OS improvement between these two therapies and the other combination therapies, except for ADT plus docetaxel (Figure 6D).

Similar to that in high-volume disease, all six therapies included showed improvements in rPFS in low-volume disease compared with ADT with or without SNA (Figure 7B). Among these therapies, triplet therapy with ADT plus enzalutamide plus docetaxel showed the most significant improvement in rPFS (HR: 0.27, 95% CrI: 0.15–0.51), followed by ADT plus enzalutamide (HR: 0.29, 95% CrI: 0.22–0.39) and ADT plus apalutamide (HR: 0.35, 95% CrI: 0.22–0.57), with a reduction in progression risks by 73%, 71%, and 65%, respectively. According to the treatment ranking analysis, both the enzalutamide triplet and doublet therapies had the highest probability of providing maximum rPFS, with SUCRAs of 0.86 and 0.85, respectively (Figure 7C). Comparing any two of these therapies, the enzalutamide triplet and doublet therapies were superior to ADT plus docetaxel or abiraterone on rPFS in low-volume disease (Figure 7D).

### 3.6 AEs

**FIGURE 8:**
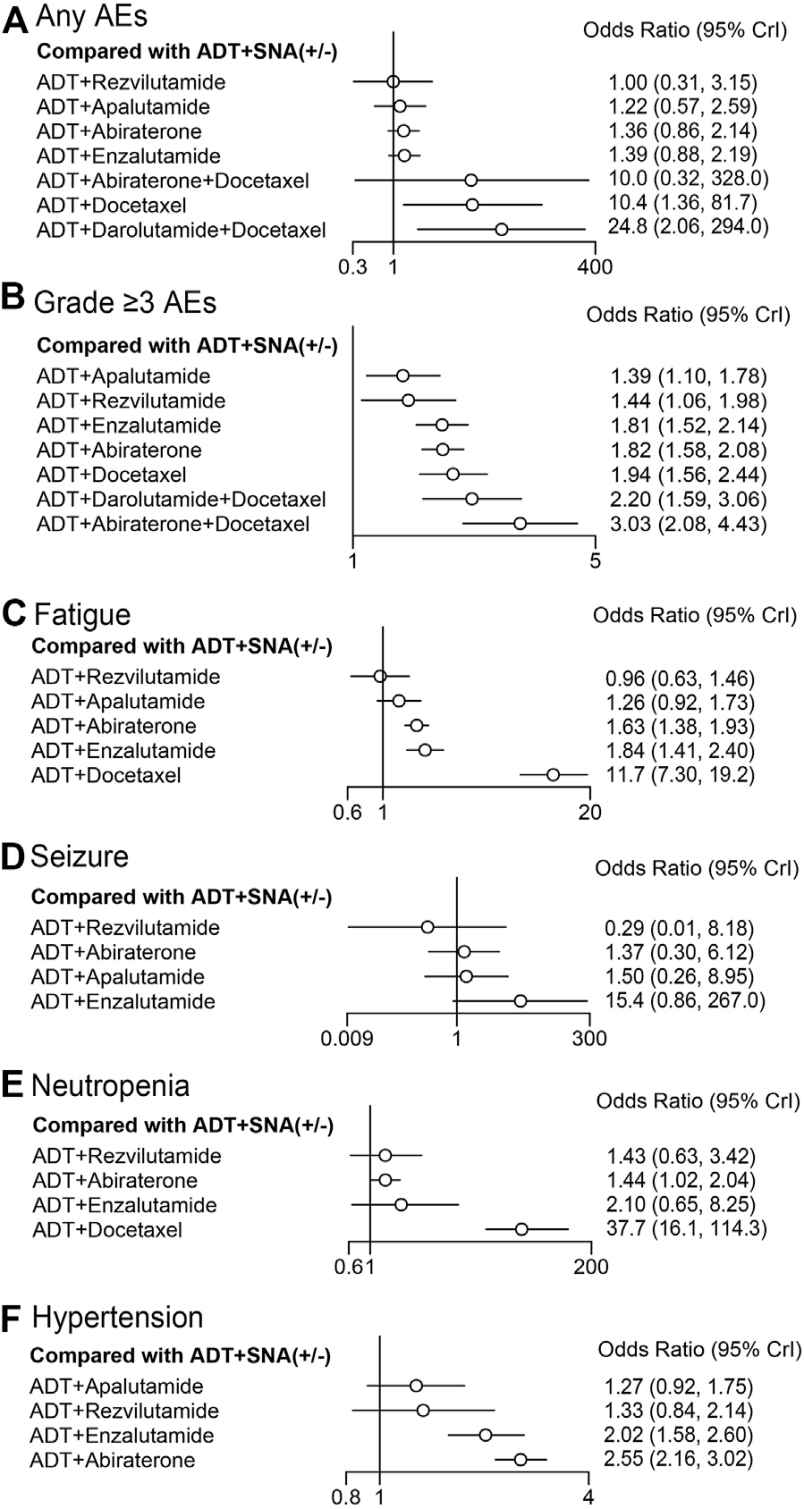
Forest plot representing a comparison of adverse events for specific therapy compared with ADT with or without SNA. OR and 95% CrI are represented. **(A)** Any AEs; **(B)** grade ≥3 AEs; **(C)** fatigue; **(D)** seizure; **(E)** neutropenia; **(F)** hypertension. ADT, androgen deprivation therapy; SNA, standard non-steroidal antiandrogen; AEs, adverse events; OR, odds ratio.

We assessed any AEs and grade ≥3 AEs among the seven combination therapies in the overall population of mHSPC using NMA. In comparison with ADT with or without SNA, none of the doublet therapies with ADT and ARTA had an increased risk of AEs. ADT plus rezvilutamide showed the lowest incidence of any AEs for mHSPC (OR: 1.00, 95% CrI: 0.31–3.15). In comparison, docetaxel-based doublet or triplet therapies significantly increased the risk of any AEs compared with ADT with or without SNA (Figure 8A). All combination therapies increased the risk of grade ≥3 AEs. ADT plus apalutamide and ADT plus rezvilutamide had a relatively lower incidence than other therapies, while docetaxel-based therapies had a high risk of grade ≥3 AEs (Figure 8B).

Four specific AEs with a high incidence were selected for further comparison among doublet therapies (Figures 8C–F). ADT plus rezvilutamide had the lowest incidence of fatigue (OR: 0.96, 95% CrI: 0.63–1.46), seizure (OR: 0.29, 95% CrI: 0.01–8.18), and neutropenia (OR: 1.43; 95% CrI: 0.63–3.42) than other doublet therapies. ADT plus enzalutamide had a relatively high incidence of fatigue (OR: 1.84, 95% CrI: 1.41–2.40), seizure (OR: 15.4, 95% CrI: 0.86–267.0), and hypertension (OR: 2.02, 95% CrI: 1.58–2.60). ADT plus docetaxel significantly increased the risks of fatigue (OR: 11.7, 95% CrI: 7.30–19.2) and neutropenia (OR: 37.7, 95% CrI: 16.1–114.3). There was a slight increase in hypertension with ADT plus apalutamide (OR: 1.27, 95% CrI: 0.92–1.75) and ADT plus rezvilutamide (OR: 1.33, 95% CrI: 0.84–2.14); however, the difference was not statistically significant. ADT plus abiraterone was associated with the highest incidence of hypertension (OR: 2.55, 95% CI: 2.16–3.02). Detailed information on results of AEs of included trials, and the pooled data for ADT plus abiraterone and ADT plus enzalutamide is shown in Supplementary Table S3B; Supplementary Figure S5.

## 4 Discussion

Several systematic reviews and NMAs have been published on systemic therapies for mHSPC, including the novel triplet therapy. Despite this, some studies have focused on evaluating the efficacy of triplet therapy or the role of docetaxel in triplet therapy, while some excluded data that was recently published. In this study, we systematically reviewed all currently available therapies for mHSPC. In addition, an NMA was performed to indirectly compare the efficacy and safety of these specific therapies especially with regard to the likelihood of providing maximum benefit in high- and low-volume disease. To the best of our knowledge, this is the first systematic review and NMA to include ADT plus rezvilutamide, as well as to indirectly compare all specific systemic therapies for mHSPC through NMA.

We pooled various current therapies into three categories in the overall population with mHSPC: ADT plus ARTA plus docetaxel, ADT plus ARTA, and ADT plus docetaxel, and evaluated their efficacies. Triplet therapy with ADT plus ARTA plus docetaxel was more effective than doublet therapy with ADT plus ARTA or ADT plus docetaxel in terms of OS and rPFS, which was consistent with previous NMA (Roy et al., 2022). As a result of different inclusion criteria or baseline characteristics across trials, especially the tumor volume, NMA or possibility ranking of specific therapies was not performed here. The high- or low-volume mHSPC was initially proposed and used as a stratification factor in the CHAARTED trial. High-volume disease was defined as the presence of visceral metastases or ≥4 bone lesions with ≥1 beyond the vertebral bodies and pelvis (Sweeney et al., 2015). Long-term survival results from that trial concluded that, with a median follow-up of 53.7 months, high-volume and low-volume control groups treated with ADT differed significantly in median OS (34.3 months in the subgroup of high-volume *versus* not reached in low-volume) (Kyriakopoulos et al., 2018). Since the distinct prognoses of high- and low-volume have also been validated in other studies, volume stratification in mHSPC has gained widespread acceptance and adopted in treatment guidelines (Kanesvaran et al., 2022; Mottet et al., 2022; Schaeffer et al., 2022). Considering that patients with the high- or low-volume disease may benefit differently from the same therapy, we stratified the data from all therapies according to patients with mHSPC with high *versus* low volume and performed NMA in the following analysis.

Efficacy rankings of therapies vary between high- and low-volume disease. For mHSPC patients with high-volume disease, triplet therapy with ADT plus ARTA plus docetaxel was shown to be more effective for OS than the doublet of ADT plus ARTA, which was consistent with the ranking in the overall population. However, docetaxel-based doublet and triplet therapy did not bring OS benefit in patients with low-volume disease compared with ADT with or without SNA, and ADT plus ARTA appears to be the only effective therapy in this subgroup.

We indirectly compared the efficacies of all current specific therapies for mHSPC in high- and low-volume disease subgroups. A previous NMA has conducted similar comparisons (Mandel et al., 2022). There was, however, no mention of the recent novel doublet therapy with ADT plus rezvilutamide and triplet therapy with ADT plus enzalutamide plus docetaxel. As far as we are aware, this is the first NMA to include ADT plus rezvilutamide. Rezvilutamide is a novel second-generation antiandrogen agent, which has been demonstrated the efficacy and safety in combination with ADT for high-volume mHSPC in the CHART trial. Interim analyses showed that ADT plus rezvilutamide improved OS (HR: 0.58, 95% CrI: 0.44–0.77) and rPFS (HR: 0.44, 95% CrI: 0.33–0.58) compared with ADT plus bicalutamide (Gu et al., 2022). Based on our NMA, ADT plus rezvilutamide was ranked first among all available doublet therapies for OS and rPFS in patients with high-volume disease.

As compared with our NMA, the improvement in OS with ADT plus abiraterone in low-volume disease in previous NMAs was more significant (HR: 0.68, 95% CrI: 0.50–0.91 *versus* HR: 0.79, 95% CrI: 0.64–0.98) (Mandel et al., 2022). The previous NMA suggested that ADT plus abiraterone showed better efficacy than the triplet therapy of ADT plus abiraterone plus docetaxel, which was inconsistent with our findings. This discrepancy may be due to the inclusion of different trials in the analysis. The previous NMA only included the LATITUDE and STAMPED G trials, while our study also included the PEACE-1 trial. In PEACE-1 trials, the OS did not significantly improve with ADT plus abiraterone plus radiotherapy (+/−) compared with ADT plus radiotherapy (+/−) in low-volume disease.

For high-volume disease, triplet therapy with ADT plus abiraterone plus docetaxel and the doublet therapy of ADT plus rezvilutamide were ranked the first two probabilities of providing maximum OS, with SUCRAs of 0.91 and 0.79, respectively. For low-volume disease, the doublet therapy of ADT plus apalutamide and ADT plus enzalutamide showed the best improvement in OS, while the triplet therapies showed no improvement compared with ADT with or without SNA. The differential efficacy of triplet therapy in high- and low-volume disease may be due to the agents in the combination. The enzalutamide triplet therapy showed no significant improvement in either subgroup, and the darolutamide triplet therapy lacked data on volume subgroups. Therefore, the pooled efficacy of triplet therapy was mainly based on the performance of ADT plus abiraterone plus docetaxel in the PEACE-1 trial. In NMA, the addition of docetaxel to ADT demonstrated no significant improvement in OS, and the addition of abiraterone had limited efficacy in low-volume disease. These two doublet therapies were both ranked last in terms of efficacy, which may lead to the fact that the triplet therapy of ADT plus abiraterone plus docetaxel, including those two agents, was inferior to the other therapies. Despite the data being integrated from multiple trials, the results of our NMA showed consistency and correlation between the efficacies of triplet and doublet therapy. This is interesting but reasonable.

Previous meta-analyses have shown that most triplet therapies of ADT plus ARTA plus docetaxel are more effective than ADT plus docetaxel (Jian et al., 2022; Yanagisawa et al., 2022). As compared with other therapies for both high-volume disease and low-volume disease, our NMA showed ADT plus docetaxel therapy to be the least effective, and was not even significantly more effective than ADT with or without SNA in patients with the low-volume disease. Other studies have also demonstrated that ADT plus ARTA is superior to ADT plus docetaxel for treating mHSPC (Wang et al., 2021; Mori et al., 2022). Therefore, the role of docetaxel in the current systemic triplet therapy remains controversial. A previous NMA suggested that, the triplet therapy of ADT plus ARTA plus docetaxel had a modest OS benefit compared with ADT plus ARTA, although without statistical significance (Roy et al., 2022). For clarification of this controversy, further head-to-head controlled trials comparing triplet therapy with ADT plus ARTA are needed.

Despite the survival advantages of triplet therapies in the overall population and subgroup of high-volume disease, the incidence of any AE or grade ≥3 AE with docetaxel-based therapies was higher than that with the doublet therapy of ADT plus ARTA. The incidence of specific AEs in triplet therapies was not compared with doublet therapies in our NMA because of the limited data. However, in our previous NMA, we found an increased risk of grade ≥3 AE in the abiraterone triplet therapy as well as hypertension in the abiraterone or darolutamide triplet therapy when compared with ADT plus docetaxel (Jian et al., 2022). Fatigue and seizure are the most common AEs associated with second-generation androgen receptor inhibitors, which might be attributable to the agent’s ability to cross the blood-brain barrier. Our NMA suggested that the risks of fatigue and seizure with rezvilutamide was lower than that with other agents, which could be attributed to the significantly lower penetration ability of rezvilutamide across the blood-brain barrier (Gu et al., 2022). Similar to the incidence of AEs with rezvilutamide in CHART trial, the incidence of fatigue and mental impairment was comparable between ADT plus docetaxel plus darolutamide and ADT plus docetaxel plus placebo in ARASENS trial (Smith et al., 2022). Preclinical studies have demonstrated that the concentration of darolutamide is significantly lower than enzalutamide (Zurth et al., 2019).

Besides balancing the survival benefits and adverse events in the treatment of mHSPC, it is important to take into account the side effects in patients with comorbidities. The overall weighted mean percentage of patients with one or more comorbidities of prostate cancer was 28.5%, with hypertension being the most common comorbidity (41.77%) followed by diabetes (11.52%), pulmonary disease (8.12%), heart failure (6.23%), and other malignancies (4.60%), cerebrovascular diseases (4.43) and renal disease (3.95%) (Vrinzen et al., 2023). These comorbidities can impact treatment decisions and may also contribute to competing mortality risks. For instance, based on our NMA, patients with preexisting hypertension may be at an increased risk of experiencing exacerbation of symptoms with the use of certain drugs like abiraterone and enzalutamide. Similarly, patients with neurological comorbidities may be at an increased risk of developing seizure with enzalutamide. In these scenarios, treatment decisions should be made with caution, and patients may benefit more from other treatment options, such as rezvilutamide. Thus, it is important to consider both the efficacy and safety of the therapy, as well as the patient’s baseline comorbidities, when making treatment decisions to ensure the best possible outcome for the patient.

This study has some limitations. First, due to the trial design, some volume stratification data were not available. Although the triplet therapy of ADT plus darolutamide plus docetaxel from the ARASENS trial has shown that the OS was improved compared with ADT plus docetaxel, data on the high- or low-volume disease subgroup were unavailable in that trial (Smith et al., 2022). Considering the relatively better efficacy of the second antiandrogen agent in low-volume disease, as well as the consistency and correlation between the efficacies of triplet therapy and doublet therapy discussed previously, it is expected that the darolutamide triplet therapy may improve the OS significantly in both high- and low-volume disease. This may change the efficacy ranking of treatment for this subgroup. Second, the data maturities of the included trials may have affected the results. Our study included updated and recent data from the included trials; however, some trials have not reached final follow-up. For example, only interim analysis of the CHART trial was included, with a median follow-up of 21.2 months (Gu et al., 2022). According to a previous NMA, different trial maturities may influence the ranking of treatments (Wenzel et al., 2022). Third, the protocols for triplet therapies are not uniform and standardized. Timing and duration of docetaxel initiation and triplet therapy vary widely across trials, resulting in varying antitumor outcomes (Jian et al., 2022). For example, in the ARCHES and TITAN trials, docetaxel was administered before ARTA, while in the well-designed PEACE-1 and ARASENS trials, all patients received six cycles of concurrent docetaxel and ARTA. Finally, the proportion of different races in trials may limit comparability between studies. The efficacies of different treatment strategies may vary among different racial subgroups. In our study, we further analyzed available data on populations of different races or regions (Supplementary Tables S7; Supplementary Figures S9–S11). In the subgroup of white population, triplet therapy with darolutamide significantly improved OS. ADT with rezvilutamide showed significant advantages in OS and rPFS in the subgroup of Asian population, and its CHART trial recruited approximately 90% of patients from China (Gu et al., 2022). However, benefitial trends in different racial subgroups were observed in these trials, although some did not reach statistical significance. This may be due to the trial design and insufficient sample size in specific subgroups.

Thus, deciding which therapy is best for mHSPC with high- or low-volume disease may be premature at this point. More triplet therapies are expected to evaluate their efficacy in mHSPC. In addition to docetaxel-based triplet therapy, the TALAPRO-3 trial (ADT plus enzalutamide plus talazoparib) and the CAPItello-281 trial (ADT plus abiraterone plus capivasertib) are ongoing (Fizazi et al., 2021; Agarwal et al., 2022). In addition, doublet therapy with ADT plus some novel ARTA has been explored. For instance, ADT plus darolutamide without docetaxel is being studied in mHSPC in the ARANOTE trial (Haresh et al., 2022). Besides, studies on ADT plus rezvilutamide in mHSPC with low-volume disease are also warranted.

## 5 Conclusion

In this systematic review and NMA, we indirectly compared the efficacy of systemic combination therapies in patients with high- or low-volume mHSPC. Triplet therapy was the best treatment option for the overall population. Triplet therapy with ADT, ARTA, and docetaxel has the greatest potential for benefiting mHSPC patients with high-volume disease. Patients with low-volume mHSPC were most likely to benefit from ADT plus ARTA. Doublet therapy was associated with fewer AEs than triplet therapy. ADT plus rezvilutamide was ranked second in efficacy following triplet therapy, with a lower risk of AEs in high-volume disease. Our findings may help clinicians determine the most personalized treatment for their patients. With more trials underway and more data available, more treatment options are expected in the future.

## Data Availability

The original contributions presented in the study are included in the article/Supplementary Material, further inquiries can be directed to the corresponding author.
