# Supplementary material for: Combination therapy for high-volume versus low-volume metastatic hormone-sensitive prostate cancer: A systematic review and network meta-analysis
Source: Front Pharmacol. 2023 Apr 20;14:1148021. doi: 10.3389/fphar.2023.1148021 (PMC10157498; doi:10.3389/fphar.2023.1148021)

Supplementary Table 1 Search strategies in this systematic review

Supplementary Table 1.1 Search strategy in Pubmed

#1	" Prostate Cancer "[Mesh]
#2	(Prostate Neoplasms[Title/Abstract]) OR (Neoplasms, Prostate[Title/Abstract]) OR (Neoplasm, Prostate[Title/Abstract]) OR (Prostate Neoplasm[Title/Abstract]) OR (Neoplasms, Prostatic[Title/Abstract]) OR (Neoplasm,Prostatic[Title/Abstract])OR (Prostatic Neoplasm[Title/Abstract]) OR (Prostate Neoplasm[Title/Abstract]) OR (Cancer, Prostate[Title/Abstract]) OR (Cancers, Prostate[Title/Abstract]) OR (Prostate Cancers[Title/Abstract]) OR (Cancer of the Prostate[Title/Abstract]) OR (Prostatic Cancer[Title/Abstract]) OR (Cancer, Prostatic[Title/Abstract]) OR (Cancers, Prostatic[Title/Abstract]) OR (Prostatic Cancers[Title/Abstract]) OR (Cancer of Prostate[Title/Abstract])
#3	#1 OR #2
#4	"Metastasis" [Mesh]
#5	(Neoplasm Metastases[Title/Abstract]) OR (Metastases, Neoplasm[Title/Abstract]) OR (Metastasis, Neoplasm[Title/Abstract]) OR (Metastase[Title/Abstract]) OR (Metastases[Title/Abstract]) OR (Metastatic [Title/Abstract])
#6	#4 OR #5
#7	"Treatment" [Mesh]
#8	(Therapeutic[Title/Abstract]) OR (Therapy[Title/Abstract]) OR (Therapies[Title/Abstract]) OR (Treatments[Title/Abstract])
#9	#7 OR #8
#10	#3 AND #6 AND #9
#11	randomized controlled trial[Publication Type] OR randomized[Title/Abstract] OR placebo[Title/Abstract]
#12	#10 AND #11

Supplementary Table 1.2 Search strategy in Embase

#1	'prostate neoplasms'/exp
#2	'prostate neoplasm':ab,ti,kw OR 'neoplasms, prostate':ab,ti,kw OR 'neoplasm, prostate':ab,ti,kw OR 'prostate neoplasm':ab,ti,kw OR 'neoplasms,prostatic':ab,ti,kw OR 'neoplasm, prostatic':ab,ti,kw OR 'prostatic neoplasm':ab,ti,kw OR 'prostate cancer':ab,ti,kw OR 'cancer, prostate':ab,ti,kw OR 'cancers, prostate':ab,ti,kw OR 'prostate cancers':ab,ti,kw OR 'cancer of the prostate':ab,ti,kw OR 'prostatic cancer':ab,ti,kw OR 'cancer, prostatic':ab,ti,kw OR 'cancers, prostatic':ab,ti,kw OR 'prostatic cancers':ab,ti,kw OR 'cancer of prostate':ab,ti,kw
#3	#1 OR #2
#4	'neoplasm metastase '/exp
#5	('neoplasm metastases':ab,ti,kw OR 'metastases, neoplasm':ab,ti,kw OR 'metastasis, neoplasm':ab,ti,kw OR 'metastase':ab,ti,kw OR 'metastases':ab,ti,kw OR 'metastasis':ab,ti,kw) AND 'metastatic':ti,ab,kw
#6	#4 OR #5
#7	' treatment'/exp

#8	' therapeutic ':ab,ti,kw OR ' therapy ':ab,ti,kw OR ' therapies ':ab,ti,kw OR ' treatments ':ab,ti,kw
#9	#7 OR #8
#10	#3 AND #6 AND #9
#11	'randomized controlled trial'/exp
#12	#10 AND #11

Supplementary Table 1.3 Search strategy in Cochrane CENTRAL

#1	MeSH descriptor: [Cancer of the Prostate] explode all trees
#2	(Prostate Neoplasms):ti,ab,kw OR (Neoplasms, Prostate):ti,ab,kw OR (Neoplasm, Prostate):ti,ab,kw OR (Prostate Neoplasm):ti,ab,kw OR (Neoplasms, Prostatic):ti,ab,kw OR (Neoplasm, Prostatic):ti,ab,kw OR (Prostatic Neoplasm):ti,ab,kw OR (Prostate Cancer):ti,ab,kw OR (Cancer, Prostate):ti,ab,kw OR (Cancers, Prostate):ti,ab,kw OR (Prostate Cancers):ti,ab,kw OR (Prostatic Cancer):ti,ab,kw OR (Cancer, Prostatic):ti,ab,kw OR (Cancers, Prostatic):ti,ab,kw OR (Prostatic Cancers):ti,ab,kw OR (Cancer of Prostate):ti,ab,kw
#3	(#1 OR #2)
#4	MeSH descriptor: [Neoplasms Metastasis] explode all trees
#5	(Neoplasm Metastases):ti,ab,kw OR (Metastases, Neoplasm):ti,ab,kw OR (Metastasis, Neoplasm):ti,ab,kw OR (Metastase):ti,ab,kw OR (Metastases):ti,ab,kw OR (Metastasis):ti,ab,kw OR (metastatic):ti,ab,kw
#6	(#4 OR #5)
#7	MeSH descriptor: [treatment] explode all trees
#8	(therapeutic):ti,ab,kw OR (therapy):ti,ab,kw OR (therapies):ti,ab,kw OR (treatments):ti,ab,kw
#9	(#7 OR #8)
#10	(#3 AND #6 AND #9)
#11	MeSH descriptor: [randomized controlled trial] explode all trees
#12	(#10 AND #11)

Supplementary Table 2 Definitions of efficacy outcomes and disease of volume in included trials in network meta-analysis

**Trial ID**	**Items**	**Definition**
**GETUG-AFU 15**	Overall survival	The time between randomization and death from any cause.
	Radiographic progression-free survival	Time from randomization to the first objective evidence of radiographic disease progression, or death, whichever occurred first. Radiographic disease progression is defined by RECTST version 1.0.
	High-volume disease	High-burden patients had either four or more bone metastases including one or more outside the vertebral body or pelvis, or any visceral metastases, or both.
	Low-volume disease	All other patients with metastases at baseline after exclusion of high-volume disease were classified as having a low-volume disease.
**CHAARTED**	Overall survival	The time from random assignment until death resulting from any cause.
	Time to clinical progression	Clinical progression was defined by increasing symptoms of bone metastases; progression according to the Response Evaluation Criteria in Solid Tumors, version 1.0; or clinical deterioration due to cancer according to the investigator's opinion.
	High-volume disease	High-burden patients had either four or more bone metastases including one or more outside the vertebral body or pelvis, or any visceral metastases, or both.
	Low-volume disease	All other patients with metastases at baseline after exclusion of high-volume disease were classified as having a low-volume disease.
**STAMPEDE arm(B, C, E)**	Overall survival	The time from random assignment until death resulting from any cause.
	Progression-free survival	The time from randomisation to the first FFS event(time from randomisation to the first of any: biochemical, lymph node, distant metastatic progression or prostate cancer death), not including biochemical progression(Biochemical progression was assessed using prostate-specific antigen (PSA) measurements which were reported at each follow-up visit.).
	High-volume disease	High-burden patients had either four or more bone metastases including one or more outside the vertebral body or pelvis, or any visceral metastases, or both.
	Low-volume disease	All other patients with metastases at baseline after exclusion of high-volume disease were classified as having a low-volume disease.

**STAMPEDE arm G**	Overall survival	The time from randomization to death from any cause.
	Progression-free survival	The time to the first of the following forms of treatment failure: progression of local, lymph-node, or distant metastases; or death from prostate cancer.
	High-volume disease	High-burden patients had either four or more bone metastases including one or more outside the vertebral body or pelvis, or any visceral metastases, or both.
	Low-volume disease	All other patients with metastases at baseline after exclusion of high-volume disease were classified as having a low-volume disease.
**ENZAMET**	Overall survival	The interval from randomization to death from any cause.
	Clinical progression-free survival	The earliest sign of radiographic progression according to the criteria of the PCWG 2 for bone lesions and RECIST, version 1.1, for soft-tissue lesions; the development of symptoms attributable to cancer progression; or the initiation of another anticancer treatment for prostate cancer.
	High-volume disease	High-volume disease was defined as the presence of visceral metastases or at least four bone lesions with at least one lesion located beyond the vertebral bodies and pelvis.
	Low-volume disease	All other patients with metastases at baseline after exclusion of high-volume disease were classified as having a low-volume disease.
**LATITUDE**	Overall survival	The time between randomization and death from any cause.
	Radiographic progression-free survival	The time from randomisation to occurrence of radiographic progression, based on the modified Prostate Cancer Working Group 2 criteria or RECIST , version 1.1.
	High-volume disease	High-volume disease was defined as presence of visceral metastases or four or more bone metastases, with at least one outside the vertebral column or pelvis.
	Low-volume disease	Disease patterns that do not meet the high-volume disease criteria are defined as low volume disease.
**ARCHES**	Overall survival	Time from randomization to death from any cause
	Radiographic progressionfree survival	Time from randomization to the first objective evidence of radiographic disease progression, or death, whichever occurred first. Radiographic disease progression is defined by RECTST version 1.1 for soft tissue disease or the appearance of 2 or more new lesions on bone scan.
	High-volume disease	High-burden patients had either four or more bone metastases including one or more outside the vertebral body or pelvis, or any visceral metastases, or both.
	Low-volume disease	All other patients with metastases at baseline after exclusion of high-volume disease were classified as having a low-volume disease.

**TITAN**	Overall survival	Time from randomization to the date of death from any cause
	Radiographic progressionfree survival	Time from randomization to first imaging-based documentation of progressive disease or death, whichever occurred first. Radiographic progressive disease: progression of softtissue lesions according to modified RECIST, version 1.1, or new bone lesions according to PCWG 2.
	High-volume disease	High-burden patients had either four or more bone metastases including one or more outside the vertebral body or pelvis, or any visceral metastases, or both.
	Low-volume disease	All other patients with metastases at baseline after exclusion of high-volume disease were classified as having a low-volume disease.
**PEACE-1**	Overall survival	The time between randomisation and death from any cause.
	Radiographic progressionfree survival	The time between randomisation and the occurrence of radiographical progression or death from any cause.Radiographical progression of soft-tissue lesions was evaluated by either CT or MRI, on the basis of RECIST version 1.1. Progression of bone lesions was assessed by bone scan according to the adapted version of Prostate Cancer Working Group 2 criteria,with no secondary bone scan required to confirm progression.
	High-volume disease	High-burden patients had either four or more bone metastases including one or more outside the vertebral body or pelvis, or any visceral metastases, or both.
	Low-volume disease	All other patients with metastases at baseline after exclusion of high-volume disease were classified as having a low-volume disease.
**CHART**	Overall survival	The time between randomisation and death from any cause.
	Radiographic progressionfree survival	Radiographic progression included progression of either soft-tissue lesion assessed by CT or MRI according to Response Evaluation Criteria in Solid Tumors (RECIST) version 1.1, or bone lesion on bone scan per the modified Prostate Cancer Working Group 3 (PCWG3) criteria.
	High-volume disease	Patients had to have high-volume disease, which was defined as presence of either four or more bone lesions on bone scan by means of [99Tc] with at least one lesion beyond the pelvis or vertebral column, or presence of visceral metastasis (excluding lymph node metastasis) by CT or MRI.
**STAMPEDE arm(C, G)**	Overall survival	The time between randomisation and death from any cause.
	Progression-free survival	The time from randomisation to the first of: new disease or progression of: distant metastases, lymph nodes or local disease; or death from prostate cancer.
**ARASENS**	Overall survival	The time between randomisation and death from any cause.

Supplementary Table 3

A. Results of Efficacy outcomes of Included Trials in Network Meta-Analysis

Trial ID	Experimental	Comparator	Overall population		High-volume disease		Low-volume disease	
			rPFS, HR (95%CI)	OS,HR(95%CI)	rPFS, HR (95%CI)	OS,HR(95%CI)	rPFS, HR (95%CI)	OS,HR(95%CI)
**GETUG-AFU 15**	ADT+Docetaxel	ADT	0.69(0.55-0.87)	0.88(0.68-1.14)	0.61(0.44-0.83)	0.78(0.56-1.09)	0.81(0.57-1.14)	1.02(0.67-1.55)
**CHAARTED**	ADT+Docetaxel	ADT	0.62(0.51-0.75) [b]	0.72(0.59-0.89)	0.53(0.42-0.67) [b]	0.63(0.50-0.79)	0.86(0.60-1.25) [b]	1.04(0.70-1.55)
**STAMPEDE arm (B, C, E)**	ADT+Docetaxel	ADT	0.69(0.59-0.81)[a]	0.81(0.69-0.95)	0.68(0.54-0.85)[a]	0.81(0.64-1.02)	0.62 (0.45–0.85) [a]	0.76(0.54-1.07)
**STAMPEDE arm G**	ADT+Abiraterone	ADT	0.45(0.37-0.54) [a]	0.61(0.49-0.79)	0.46(0.36-0.58)[a]	0.60(0.46-0.78)	0.40(0.28-0.58) [a]	0.64(0.42-0.97)
**ENZAMET**	ADT+Enzalutamide	ADT+SNA	0.40(0.33-0.49) [b]	0.67(0.52-0.86)	0.45(0.36-0.57) [b]	0.80(0.59-1.07)	0.30(0.22-0.43)[b]	0.43(0.26-0.72)
	ADT+Enzalutamide+ Docetaxel	ADT+SNA+ Docetaxel	0.48(0.37-0.62) [b]	0.9(0.62-1.31)	0.51(0.38-0.69)[b]	0.97(0.64-1.46)	0.37（0.20-0.67)[b]	0.65(0.25-1.71)
**LATITUDE**	ADT+Abiraterone	ADT	0·47(0·39–0·55)	0·66(0·56−0·78)	0.46(0.39-0.54)	0.62(0.52-0.74)	0.59(0.40-0.85)	0.72(0.47-1.10)
**ARCHES**	ADT+Enzalutamide	ADT	0.39(0.30-0.50)	0.66 (0.53-0.81)	0.43(0.33-0.57)	0.66(0.52-0.83)	0.25(0.14-0.46)	0.66(0.43-1.03)
	ADT+Enzalutamide+ Docetaxel	ADT+Docetaxel	0.52(0.30-0.89)	0.74(0.46-1.20)	NA	NA	NA	NA
**TITAN**	ADT+Apalutamide	ADT	0.49(0.40–0.61)	0.65(0.53-0.79)	0.53(0.41-0.67)	0.70(0.56-0.88)	0.36(0.22-0.57)	0.52（0.35-0.79）
	ADT+Apalutamide+ Docetaxel	ADT+Docetaxel	0.47(0.22-1.01）	1.12(0.59-2.12）	NA	NA	NA	NA
**PEACE-1**	ADT+Abiraterone+ RT(+/-)	ADT+RT(+/-)	0.54(0.46-0.64) [d]	0·82(0·69-0·98) [c]	0.51(0.42-0.63) [d]	0.77(0.62-0.96) [c]	0.59(0.45-0.77) [d]	0.93(0.69-1.28) [c]
	ADT+Abiraterone+ Docetaxel+RT(+/-)	ADT+Docetaxel +RT(+/-)	0.50(0..39-0.61) [d]	0·75(0·59-0·95) [c]	0.47(0.36-0.60)[d]	0.72(0.55-0.95)[c]	0.58(0.38-0.87) [d]	0.83(0.50-1.39) [c]
**CHART**	ADT+Rezvilutamide	ADT+SNA	0.44(0.33-0.58)	0.58(0.44-0.77)	0.44(0.33-0.58)	0.58(0.44-0.77)	NA	NA
**STAMPEDE arm**	ADT+Abiraterone	ADT+Docetaxel	0.65(0.48–0.88) [a]	1.16(0.82-1.65)	NA	NA	NA	NA

**(C, G)**								
**ARASENS**	ADT+Darolutamide+Docetaxel	ADT+Docetaxel	NA	0.68(0.57-0.80)	NA	NA	NA	NA

RT:Radiotherapy; SNA: standard nonsteroidal antiandrogen (bicalutamide, nilutamide or flutamide); NA: Not available.

a. progression-free survival

b.Clinical Progression-free survival

c. For comparison, the value has been transferred from 95.1%CI to 95%CI

d. For comparison, the value has been transferred from 99.9%CI to 95%CI

B. Results of adverse events of Included Trials in Network Meta-Analysis

Trial ID	Arms	Total number of patients (n)	Adverse event of any grade	Grade ≥3 treatment-emergent adverse event (n,%)	Hypertension (n,%)	Fatigue (n,%)	Neutropenia (n,%)	Seizure (n,%)
GETUG-AFU 15	ADT+Docetaxel	189	NA	NA	NA	140(74)	94 (50)	NA
	ADT	186	NA	NA	NA	37(20)	5 (3)	NA
STAMPEDE arm (B, C, E)	ADT+Docetaxel	362	362(100)	141(39)	NA	NA	NA	NA
	ADT	724	703(97)	179(25)	NA	NA	NA	NA
STAMPEDE arm G	ADT+Abiraterone	948	943 (99)	443(47)	299(32)	648(68)	73（8）	4(0)
	ADT	960	950 (99)	315(33)	132(14)	552(58)	47（5）	3(0)
ENZAMET	ADT+Enzalutamide	563	563(100)	321(57)	235(42)	NA	NA	7(1)
	ADT+SNA	558	548(98)	241(43)	189(34)	NA	NA	0(0)
LATITUDE	ADT+Abiraterone	597	558(93)	374(63)	243(41)	84(14)	9(2)	NA
	ADT	602	557(93)	287(48)	144(24)	90(15)	12(2)	NA
ARCHES	ADT+Enzalutamide	572	520 (91)	254(44)	82(14)	184(32)	8(1)	NA
	ADT	574	504 (88)	172(30)	39(7)	118(21)	4(1)	NA
TITAN	ADT+Apalutamide	524	510 (97)	279(53)	102(19)	107(20)	NA	3(1)
	ADT	527	510 (97)	237(45)	84(16)	89(17)	NA	2(0)
PEACE-1	ADT+Abiraterone+RT(+/)	226	226(100)	149(66)	NA	86(36)	NA	NA
	ADT+RT(+/-)	237	233(98)	97(41)	NA	61(26)	NA	NA
	ADT+Docetaxel+ Abiraterone+RT(+/-)	347	346 (100)	217(63)	NA	244(99)	NA	NA
	ADT+Docetaxel+RT(+/-)	350	349 (100)	181(52)	NA	257(73)	NA	NA
CHART	ADT+Rezvilutamide	323	317(98)	166(51)	46(14)	52(16)	14(4)	0(0)
	ADT+ SNA	324	318(98)	137(42)	36(11)	54(17)	10(3)	1(0)

**STAMPEDE arm(C, G)**	ADT+Abiraterone	373	370(99)	180(48)	NA	NA	NA	NA
	ADT+Docetaxel	172	172(100)	86(50)	NA	NA	NA	NA
**ARASENS**	ADT+Darolutamide+Docetaxel	652	649(100)	458(70)	85(13)	216(33)	256(39)	4(0)
	ADT+Docetaxel	650	643(99)	439(68)	59(9)	214(33)	252(39)	1(0)

RT:Radiotherapy; SNA: standard nonsteroidal antiandrogen (bicalutamide, nilutamide or flutamide); NA: Not available.

Supplementary Table 4 Detailed Assessment of Risk of Bias in Included Trials in Network Meta-Analysis

**Type of bias**	**Selection bias**		**Performance bias**	**Detection bias**	**Attrition bias**	**Reporting bias**	**Bias from other sources**	
**Fields in Tools**	**Sequence generation**	**Allocation concealment**	**Blinding of participants and personnel**	**Blinding of outcome assessment**	**Incomplete outcome data**	**Selective outcome reporting**	**Baseline for the intervention and control groups**	**Source of funding**
**GETUG-AFU 15**	"Randomisation was done by a clinical research organization…" **comment: unclear**	"Patients were randomly allocated in a 1:1 ratio to receive…" **comment: unclear**	"In this randomised, open-label, phase 3 trial…" **comment: high risk**	"Patients, physicians, and data analysts were not masked to treatment allocation." **comment: unclear**	Missing data had little effect on the effect values. **comment: low risk**	The study is in accordance with the study protocol registered with ClinicalTrial. (NCT00104715) **comment: low risk**	The characteristics of the intervention and control groups were similar at baseline. **comment: low risk**	"The sponsors of the study had no role in study design, data collection, data analysis, data interpretation, or writing of the report." **comment: low risk**
**CHAARTED**	"this multicenter, randomized, open-label, phase III National Cancer	"Patients were randomly assigned to ADT alone or to combination therapy…" **comment:**	"this multicenter, randomized, open-label, phase III National Cancer Institute study led by…" **comment:**	"this multicenter, randomized, open-label, phase III National Cancer Institute study led by…"	The number and causes of missing persons were similar between the groups.	The study is in accordance with the study protocol registered with ClinicalTrial. (NCT00309985)	The characteristics of the intervention and control groups were similar at baseline.	"Sanofi donated the docetaxel for early use …but had no role in the design of the protocol...." **comment:**

	Institute study led by…" **comment: unclear**	**unclear**	**high risk**	**comment: unclear**	**comment: low risk**	**comment: low risk**	**comment: low risk**	**low risk**
**STAMPEDE arm(B, C, E)**	"Patients were randomised centrally using a computerised algorithm…" **comment: low risk**	"Minimisation with a random element of 80% was used, stratifying for hospital…" **comment: unclear**	"Masking to treatment allocation was considered impracticable …" **comment: high risk**	Accumulating data were reviewed by an Independent Data Monitoring Committee, guided by lack-of-benefi t stopping guidelines. **comment: low risk**	Missing data had little effect on the effect values. **comment: low risk**	The study is in accordance with the study protocol registered with ClinicalTrial. (NCT00268476) **comment: low risk**	The characteristics of the intervention and control groups were similar at baseline. **comment: low risk**	"Pfi zer, Novartis, and Sanofi -Aventis approved the initial and amended trial design and participated in discussions on the progress of the trial." **comment: unclear**
**STAMPEDE arm G**	"Randomization was performed centrally by telephone with the use of a computerized algorithm…" **Comment: low risk**	"Minimization with a random element of 80% was used, with stratification according to randomizing center…" **comment: unclear**	"The comparison was open-label, because masking of the treatment assignment was deemed impracticable." **comment: high risk**	"processed data that were released by the independent data monitoring committee and trial steering committee were available to all the coauthors."	Missing data had little effect on the effect values. **comment: low risk**	The study is in accordance with the study protocol registered with ClinicalTrial. (NCT00268476) **comment: low risk**	The characteristics of the intervention and control groups were similar at baseline. **comment: low risk**	"Janssen approved the design for this comparison … Representatives from Janssen were invited to comment on the manuscript."

				**Comment: low risk**				**comment: unclear**
**ENZAMET**	"In this open-label, randomized, phase 3 trial,…" **comment: unclear**	"The central randomization system implemented minimization with a random component" **Comment: low risk**	"this multinational, openlabel, randomized, phase 3 trial…" **Comment: high risk**	"An independent data and safety monitoring committee reviewed the progress and results of the trial. **Comment: low risk**	Missing data had little effect on the effect values. **comment: low risk**	The study is in accordance with the study protocol registered with ClinicalTrial. (NCT02446405) **comment: low risk**	The characteristics of the intervention and control groups were similar at baseline. **comment: low risk**	"representativ--es of the company …but were not otherwise involved in any aspects of the trial design, data accrual, data analysis, or manuscript preparation" **Comment: low risk**
**LATITUDE**	"randomly assigned (1:1) to receive ADT with abiraterone acetate plus prednisone or ADT with matching placebos, using a computer-gen	"A unique identification number and treatment number for each patient were generated by a centralised interactive web response system." **comment: low risk**	"The randomisation codes were maintained within the interactive web response system and treatment allocations were masked to investigators, patients, and study personnel until study completion,“	" The randomisation codes were maintained within the interactive web response system and treatment allocations were masked to investigators,	The number and causes of missing persons were similar between the groups. **comment: low risk**	The study is in accordance with the study protocol registered with ClinicalTrial. (NCT01715285) **comment: low risk**	The characteristics of the intervention and control groups were similar at baseline. **comment: low risk**	Funding:Janssen Research & Development. **Comment: unclear**

	erated randomisation schedule." **comment: low risk**		**comment: low risk**	patients, and study personnel until study completion," **comment: low risk**				
**ARCHES**	"Randomization was performed centrally by telephone with the use of a computerized algorithm" **comment: low risk**	"Randomization was performed centrally by telephone with the use of a computerized algorithm" **Comment: low risk**	Masking: Quadruple (Participant, Care Provider, Investigator, Outcomes Assessor) **Comment: low risk**	"processed data that were released by the independent data monitoring committee …" **Comment: low risk**	Missing data had little effect on the effect values. **comment: low risk**	The study is in accordance with the study protocol registered with ClinicalTrial. (NCT02677896) **comment: low risk**	The characteristics of the intervention and control groups were similar at baseline. **comment: low risk**	"Further funding for the platform was provided by Astellas Pharma,… " **Comment: unclear**
**TITAN**	"The investigators, patients, trial-site personnel, and sponsor trial team were una ware of the randomization codes until	"The investigators, patients, trial-site personnel, and sponsor trial team were una ware of the randomization codes until trial completion" **Comment: low risk**	"The investigators, patients, trial-site personnel, and sponsor trial team were una ware of the randomization codes until trial completion" **Comment: low risk**	"An independent datamonitoring committee was commissioned by the sponsor to monitor safety and efficacy before unblinding and to make recommendation	The number and causes of missing persons were similar between the groups. **comment: low risk**	The study is in accordance with the study protocol registered with ClinicalTrial. (NCT02489318) **comment: low risk**	The characteristics of the intervention and control groups were similar at baseline. **comment: low risk**	"Funded by Janssen Research and Development" **Comment: unclear**

	trial completion” **Comment: low risk**			s regarding trial conduct.” **Comment: low risk**				
**PEACE-1**	“This randomisation process was performed via the Tenalea autonomous softwa re,” **Comment: low risk**	“Randomisation was done using a minimisation algorithm,” **Comment: unclear**	“an open-label, randomised, active-controlled, phase 3 study” **Comment: high risk**	“Steering and Independent Data Monitoring Committees roles and members are described in the appendix” **Comment: low risk**	Missing data had little effect on the effect values. **comment: low risk**	The study is in accordance with the study protocol registered with ClinicalTrial. (NCT01957436) **comment: low risk**	The characteristics of the intervention and control groups were similar at baseline. **comment: low risk**	“The funders of the study had no role in study design, data collection, data analysis, data interpretation, or writing of the report. ” **Comment: low risk**
**CHART**	“Patients were randomly assigned… via an interactive response technology system with a block size of four.” **comment: low risk**	“The investigators at each site registered patients and assigned them according to the randomisation sequences generated. ” **comment: low risk**	“CHART is a randomised, open-label, phase 3 study…” **comment: high risk**	“…and the data were reviewed by an independent data monitoring.” **comment: low risk**	The number and causes of missing persons were similar between the groups. **comment: low risk**	The study is in accordance with the study protocol registered with ClinicalTrial. （NCT03520478） **comment: low risk**	The characteristics of the intervention and control groups were similar at baseline. **comment: low risk**	“Funding: Jiangsu Hengrui Pharmaceuticals .” **Comment: unclear**
**STAMPEDE arm(C, G)**	“Patients were randomised	“Patients were randomised	“The comparison was open-label,…”	“Imaging scans after baseline	Missing data had little effect	The study is in accordance with	The characteristics	“The trial was sponsored by the

	centrally using a computerised algorithm…" **comment: low risk**	centrally using minimisation with a random element…" **Comment: unclear**	**comment: high risk**	were at the investigator's discretion." **Comment: unclear**	on the effect values. **comment: low risk**	the study protocol registered with ClinicalTrial. (NCT00268476) **comment: low risk**	of the intervention and control groups were similar at baseline. **comment: low risk**	UK Medical Research Council (MRC) and conducted..." **Comment: unclear**
**ARASENS**	"In this international, randomized, doubleblind, placebo-controlled trial..." **Comment: unclear**	"Participants Randomized by Center" **comment: low risk**	Masking: Quadruple (Participant, Care Provider, Investigator, Outcomes Assessor) **comment: low risk**	"An independent data and safety monitoring board reviewed unblinded safety and efficacy data throughout the trial." **comment: low risk**	Missing data had little effect on the effect values. **comment: low risk**	The study is in accordance with the study protocol registered with ClinicalTrial. (NCT02799602) **comment: low risk**	The characteristics of the intervention and control groups were similar at baseline. **comment: low risk**	"Funded by Bayer and Orion Pharma" **Comment: unclear**

Supplementary Table 5 Certainty of the evidence for each outcomes based on the GRADE approach

**Outcomes**	**Certainty of the evidence (GRADE)**
Overall survival	⊕⊕⊕⊖<br>**Moderate**[1]
Radiographic progression-free survival	⊕⊕⊕⊖<br>**Moderate**[1]
Adverse event of any grade	⊕⊕⊖⊖<br>**Low** [1,2]
grade 3-4 treatment-emergent adverse event	⊕⊕⊕⊖<br>**Moderate**[1]
Hypertension	⊕⊕⊕⊖<br>**Moderate**[1]
Fatigue	⊕⊕⊕⊖<br>**Moderate**[1]
Neutropenia	⊕⊕⊖⊖<br>**Low** [1,2]
Seizure	⊕⊕⊖⊖<br>**Low** [1,2]

[1]Study limitation(risk of bias)

[2]Imprecision

Certainty of evidence in assessing outcomes in five respects:

1. Study limitation(risk of bias)

**Author's adjustment**	**Individual studies**	**Studies**
**Low risk of bias**	All 7 items were assessed as low risk of bias	
**Unclear**	One or more items were assessed as unclear	ARCHES,LATITUDE,TITAN, ARASENS
**High risk of bias**	One or more items were assessed as high risk of bias	CHAARTED,CHART,ENZAMET,PEACE-1 GETUG-AFU 15, STAMPEDE arm(B,C,E), STAMPEDE arm G, STAMPEDE arm(C, G)

SO this aspect affect the assessment of the level of evidence.

2. Indirectness: The inclusion population, interventions and outcome assessment in the text are equivalent to the researcher concerns in the experimental design. Therefore this aspect does not affect the assessment of the level of evidence.

3. Inconsistency: Firstly, the baseline data were similar across studies. Secondly, when trials of the same type were combined, we excluded those with large differences to maintain I^2 ≤50%. Therefore, this aspect had little impact on the assessment of the level of evidence.

4. Imprecision: The interval between results was too wide or there were studies with very small sample

sizes. So this aspect affects the assessment of the level of evidence.

5. Publication bias: The study report is consistent with the registered study protocol. Therefore this aspect does not affect the assessment of the level of evidence.

GRADE Working Group grades of evidence

High certainty: We are very confident that the true effect lies close to that of the estimate of the effect

Moderate certainty: We are moderately confident in the effect estimate; the true effect is likely to be close to the estimate of the effect, but there is a possibility that it is substantially different

Low certainty: Our confidence in the effect estimate is limited; the true effect may be substantially different from the estimate of the effect

Very low certainty: We have very little confidence in the effect estimate; the true effect is likely to be substantially different from the estimate of effect

Supplementary Table 6 Efficacy results for trials included in Network Meta-analysis at secondary endpoints.

Trial ID	Experimental	Comparator	Time to next skeletal-related event		Time to PSA progression		Time to initiation of new anti-prostate cancer therapy		Time to castration resistance	
			High-volume disease, HR(95%CI)	Low-volume disease, HR(95%CI)	High-volume disease, HR(95%CI)	Low-volume disease, HR(95%CI)	High-volume disease, HR(95%CI)	Low-volume disease, HR(95%CI)	High-volume disease, HR(95%CI)	Low-volume disease, HR(95%CI)
**CHART**	ADT+Rezvilutamide	ADT+SNA	0.65(0.50-0.84)	NA	0.21(0.16-0.27)	NA	0·33 (0·26-0·41)	NA	NA	NA
**ARCHES**	ADT+Enzalutamide	ADT+SNA	0.59(0.37-0.95)	0.25(0.07-0.91)	0.22(0.16-0.32)	0.08(0.03-0.20)	0.27(0.18-0.40)	0.39(0.18-0.82)	0.32(0.24-0.41)	0.18(0.10-0.32)
**CHAARTED**	ADT+Docetaxel	ADT	NA	NA	NA	NA	NA	NA	0.58(0.47-0.71)	0.70(0.50-0.96)

SNA: standard nonsteroidal antiandrogen (bicalutamide, nilutamide or flutamide); NA: Not available. ADT: androgen deprivation therapy.

Supplementary Table 7 Relevant data on races.

Supplementary Table 7.1 Efficacy results for trials included in Network Meta-analysis.

Trial ID	Experimental	Comparator	White		Asian		Black	
			OS,HR (95%CI)	rPFS,HR(95%CI)	OS,HR (95%CI)	rPFS,HR(95%CI)	OS,HR (95%CI)	rPFS,HR(95%CI)
**CHAARTED**	ADT+Docetaxel	ADT	0.72(0.58-0.89)	NA	NA	NA	NA	NA
**ENZAMET**	ADT+Enzalutamide	ADT+SNA	0.67(0.52–0.86)[a]	0.40(0.33–0.49)[a]	NA	NA	NA	NA
**LATITUDE**	ADT+Abiraterone	ADT	0.64(0.53-0.78)[b]	NA	0.68(0.44-1.04)	NA	NA	NA
**TITAN**	ADT+Apalutamide	ADT	0.75(0.52-1.07)[c]	0.43(0.28-0.66)[c]	NA	NA	NA	NA
**CHART**	ADT+Rezvilutamide	ADT+SNA	0.85(0.39-1.87)	0.87(0.39-1.96)	0.55(0.40-0.74)	0.40(0.30-0.54)	NA	NA
**ARASENS**	ADT+Darolutamide+Docetaxel	ADT+Docetaxel	0.63(0.50-0.79)	NA	0.84(0.62-1.14)	NA	0.46(0.20-1.08)	NA

SNA: standard nonsteroidal antiandrogen (bicalutamide, nilutamide or flutamide); NA: Not available. ADT: androgen deprivation therapy.

a.Includes Australia, Canada, Ireland, New Zealand, United Kingdom and United States.

b.Includes Eastern Europe and Western Europe.

c. Includes North America and European Union.

Supplementary Table 7.2 Number of people for trials included in Network Meta-analysis.

Trial ID	Experimental	Comparator	White		Asian		Black	
			Experimental (no. of patients)	Comparator (no. of patients)	Experimental (no. of patients)	Comparator (no. of patients)	Experimental (no. of patients)	Comparator (no. of patients)
**CHAARTED**	ADT+Docetaxel	ADT	344	330	NA	NA	39	37
**ENZAMET**	ADT+Enzalutamide	ADT+SNA	563 [a]	562 [a]	NA	NA	NA	NA
**LATITUDE**	ADT+Abiraterone	ADT	369 [b]	379 [b]	124	121	NA	NA
**TITAN**	ADT+Apalutamide	ADT	173 [c]	173 [c]	NA	NA	NA	NA
**CHART**	ADT+Rezvilutamide	ADT+SNA	31	32	295	296	NA	NA
**ARASENS**	ADT+Darolutamide+Docetaxel	ADT+Docetaxel	345	333	230	245	NA	NA

SNA: standard nonsteroidal antiandrogen (bicalutamide, nilutamide or flutamide); NA: Not available. ADT: androgen deprivation therapy.

a.Includes Australia, Canada, Ireland, New Zealand, United Kingdom and United States.

b.Includes Eastern Europe and Western Europe.

c. Includes North America and European Union.

Supplementary Figure 1. Assessment of risk of bias of included trials. (A) Risk of bias summary for each RCT assessed according to the methods recommended by the Cochrane Collaboration. The green positive sign, low risk of bias; the red negative sign, high risk of bias; the yellow question mark, unclear risk of bias; (B) Risk of bias graph about each risk of bias item illustrated as percentage across all selected RCTs.

A.

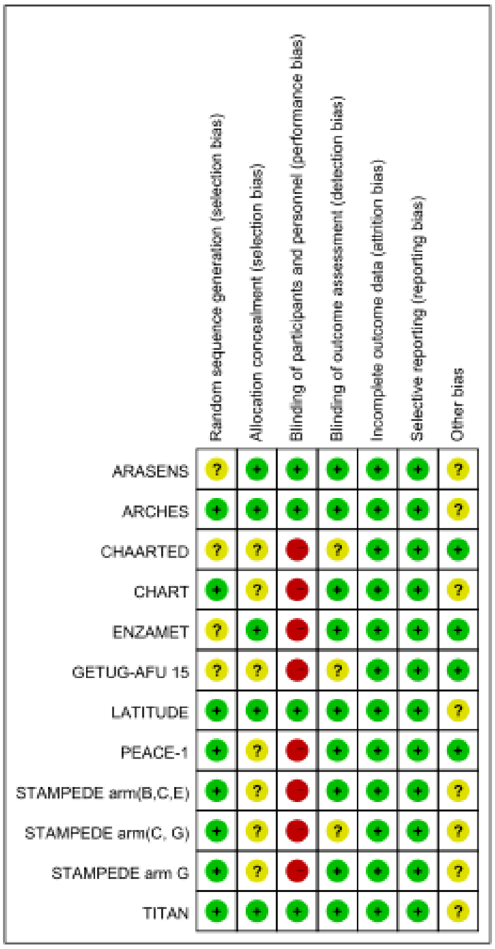

B.

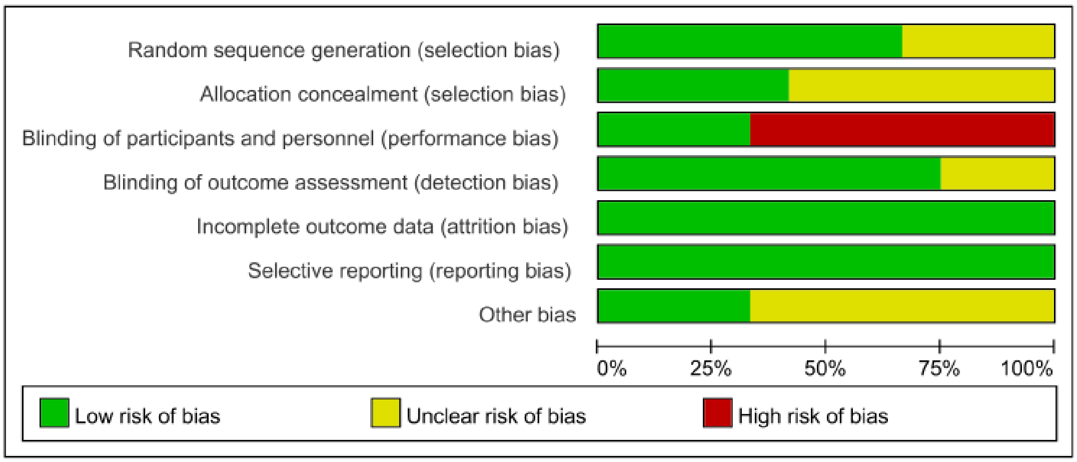

Supplementary Figure 2 Assessment of homogeneity in overall population

A. ADT plus ARAT and docetaxel versus ADT plus docetaxel on OS.

Study or Subgroup	log[Hazard Ratio]	SE	Weight	Hazard Ratio IV, Fixed, 95% CI
ARASENS	-0.3926	0.0865	52.4%	0.68 [0.57, 0.80]
ARCHES	-0.2971	0.2446	6.6%	0.74 [0.46, 1.20]
ENZAMET	-0.104	0.1908	10.8%	0.90 [0.62, 1.31]
PEACE-1	-0.2895	0.1215	26.6%	0.75 [0.59, 0.95]
TITAN	0.1119	0.3263	3.7%	1.12 [0.59, 2.12]
**Total (95% CI)**			**100.0%**	**0.73 [0.65, 0.83]**

Heterogeneity: Chi² = 3.78, df = 4 (P = 0.44); I² = 0%
Test for overall effect: Z = 4.94 (P < 0.00001)

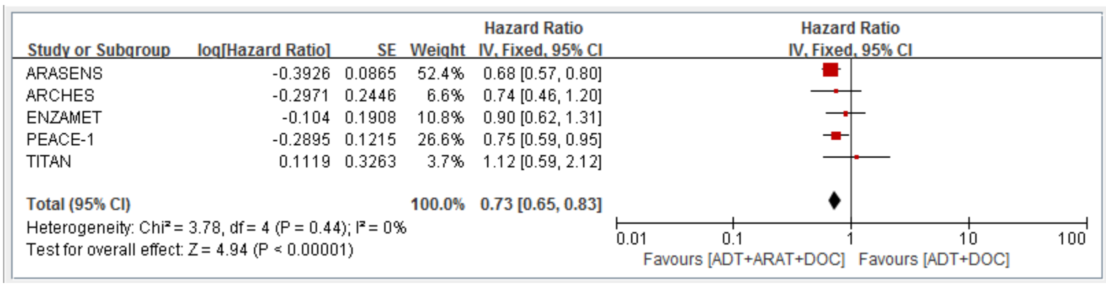

B. ADT plus ARAT and docetaxel versus ADT plus docetaxel on rPFS

Study or Subgroup	log[Hazard Ratio]	SE	Weight	Hazard Ratio IV, Fixed, 95% CI
ARCHES	-0.6603	0.2774	8.4%	0.52 [0.30, 0.89]
ENZAMET	-0.7361	0.1317	37.4%	0.48 [0.37, 0.62]
PEACE-1	-0.718	0.1141	49.9%	0.49 [0.39, 0.61]
TITAN	-0.7521	0.3888	4.3%	0.47 [0.22, 1.01]
**Total (95% CI)**			**100.0%**	**0.49 [0.42, 0.57]**

Heterogeneity: Chi² = 0.07, df = 3 (P = 1.00); I² = 0%
Test for overall effect: Z = 8.95 (P < 0.00001)

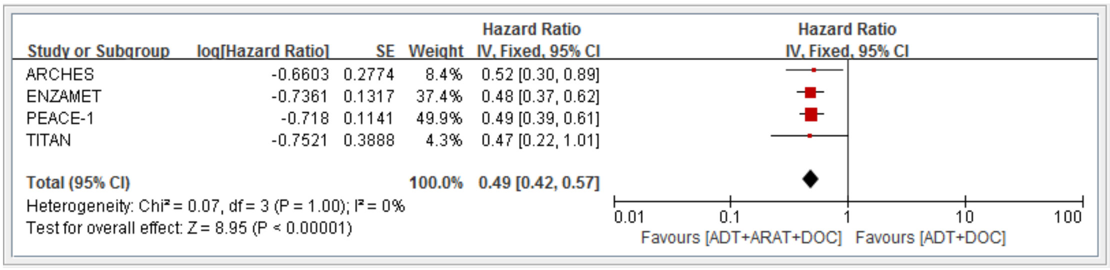

C. ADT plus ARAT versus ADT with or without SNA on OS

Study or Subgroup	log[Hazard Ratio]	SE	Weight	Hazard Ratio IV, Fixed, 95% CI
ARCHES	-0.4228	0.1082	13.7%	0.66 [0.53, 0.81]
CHART	-0.5412	0.1428	7.9%	0.58 [0.44, 0.77]
ENZAMET	-0.4024	0.1283	9.7%	0.67 [0.52, 0.86]
LATITUDE	-0.4141	0.0845	22.4%	0.66 [0.56, 0.78]
PEACE-1	-0.1956	0.0895	20.0%	0.82 [0.69, 0.98]
STAMPEDE arm G	-0.4745	0.1218	10.8%	0.62 [0.49, 0.79]
TITAN	-0.4353	0.1018	15.5%	0.65 [0.53, 0.79]
**Total (95% CI)**			**100.0%**	**0.68 [0.63, 0.73]**

Heterogeneity: Chi² = 6.70, df = 6 (P = 0.35); I² = 10%
Test for overall effect: Z = 9.75 (P < 0.00001)

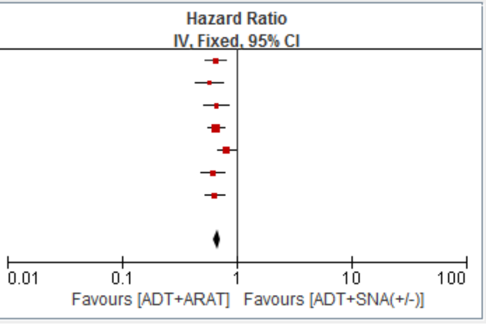

D. ADT plus ARAT versus ADT with or without SNA on rPFS

Study or Subgroup	log[Hazard Ratio]	SE	Weight	Hazard Ratio IV, Fixed, 95% CI
ARCHES	-0.9486	0.1303	8.8%	0.39 [0.30, 0.50]
CHART	-0.8267	0.1439	7.2%	0.44 [0.33, 0.58]
ENZAMET	-0.911	0.1008	14.7%	0.40 [0.33, 0.49]
LATITUDE	-0.7697	0.0877	19.4%	0.46 [0.39, 0.55]
PEACE-1	-0.6114	0.0842	21.0%	0.54 [0.46, 0.64]
STAMPEDE arm G	-0.8052	0.0964	16.0%	0.45 [0.37, 0.54]
TITAN	-0.7053	0.1077	12.9%	0.49 [0.40, 0.61]
**Total (95% CI)**			**100.0%**	**0.46 [0.43, 0.50]**

Heterogeneity: Chi² = 8.02, df = 6 (P = 0.24); I² = 25%
Test for overall effect: Z = 20.05 (P < 0.00001)

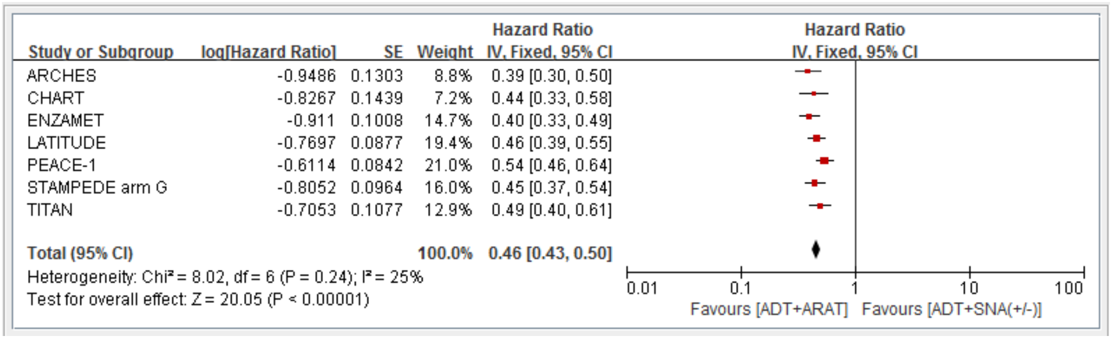

E. ADT plus docetaxel versus ADT with or without SNA on OS

Study or Subgroup	log[Hazard Ratio]	SE	Weight	Hazard Ratio IV, Fixed, 95% CI
CHAARTED	-0.3221	0.1049	30.4%	0.72 [0.59, 0.89]
GETUG-AFU 15	-0.1273	0.1318	19.3%	0.88 [0.68, 1.14]
STAMPEDE arm (B,C,E)	-0.2112	0.0816	50.3%	0.81 [0.69, 0.95]
**Total (95% CI)**			**100.0%**	**0.80 [0.71, 0.89]**

Heterogeneity: Chi² = 1.43, df = 2 (P = 0.49); I² = 0%
Test for overall effect: Z = 3.95 (P < 0.0001)

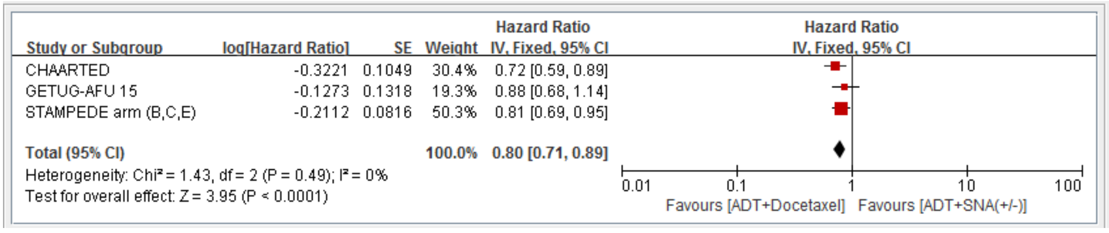

F. ADT plus docetaxel versus ADT with or without SNA on rPFS

Study or Subgroup	log[Hazard Ratio]	SE	Weight	Hazard Ratio IV, Fixed, 95% CI
CHAARTED	-0.4805	0.0984	31.3%	0.62 [0.51, 0.75]
GETUG-AFU 15	-0.3685	0.117	22.2%	0.69 [0.55, 0.87]
STAMPEDE arm (B,C,E)	-0.3692	0.0808	46.5%	0.69 [0.59, 0.81]
**Total (95% CI)**			**100.0%**	**0.67 [0.60, 0.74]**

Heterogeneity: Chi² = 0.88, df = 2 (P = 0.64); I² = 0%
Test for overall effect: Z = 7.33 (P < 0.00001)

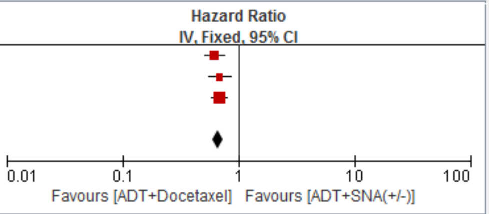

Supplementary Figure 3 Assessment of homogeneity in high-volume disease

A. ADT plus ARAT and docetaxel versus ADT plus docetaxel on OS in patients with high-volume disease.

Study or Subgroup	log[Hazard Ratio]	SE	Weight	Hazard Ratio IV, Fixed, 95% CI
ENZAMET	-0.0339	0.2104	30.5%	0.97 [0.64, 1.46]
PEACE-1	-0.3246	0.1394	69.5%	0.72 [0.55, 0.95]
**Total (95% CI)**			**100.0%**	**0.79 [0.63, 0.99]**

Heterogeneity: Chi² = 1.33, df = 1 (P = 0.25); I² = 25%
Test for overall effect: Z = 2.03 (P = 0.04)

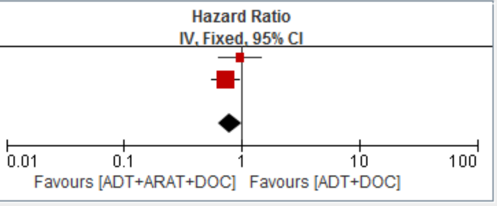

B. ADT plus ARAT and docetaxel versus ADT plus docetaxel on rPFS in patients with high-volume disease.

Study or Subgroup	log[Hazard Ratio]	SE	Weight	Hazard Ratio IV, Fixed, 95% CI
ENZAMET	-0.6693	0.1522	42.3%	0.51 [0.38, 0.69]
PEACE-1	-0.7662	0.1303	57.7%	0.46 [0.36, 0.60]
**Total (95% CI)**			**100.0%**	**0.48 [0.40, 0.59]**

Heterogeneity: Chi² = 0.23, df = 1 (P = 0.63); I² = 0%
Test for overall effect: Z = 7.33 (P < 0.00001)

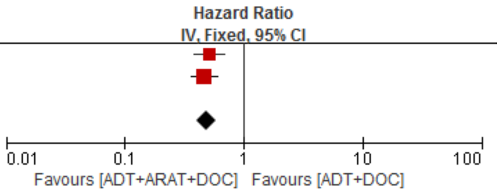

C. ADT plus ARAT versus ADT with or without SNA on OS in patients with high-volume disease.

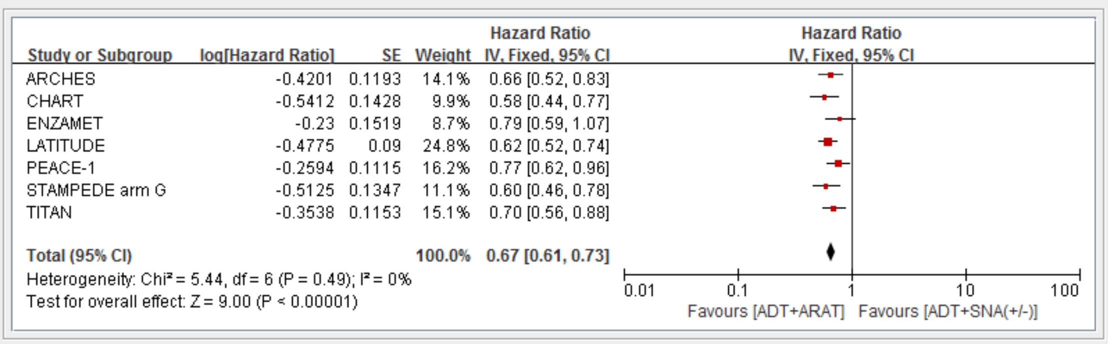

D. ADT plus ARAT versus ADT with or without SNA on rPFS in patients with high-volume disease.

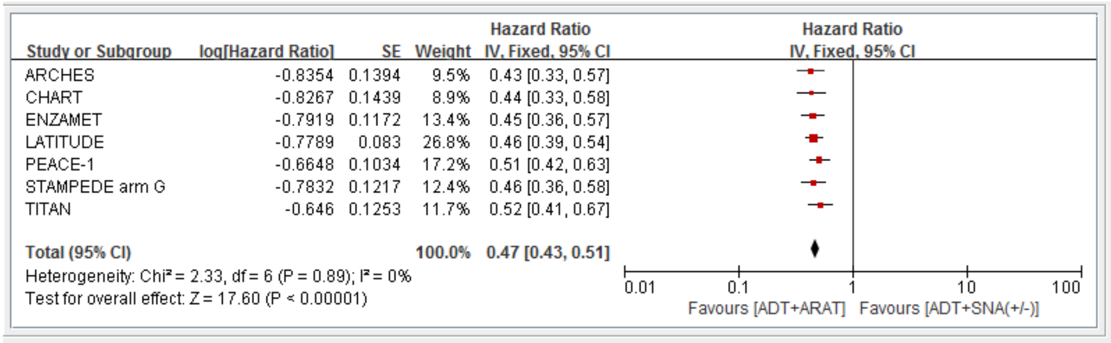

E. ADT plus docetaxel versus ADT with or without SNA on OS in patients with high-volume disease.

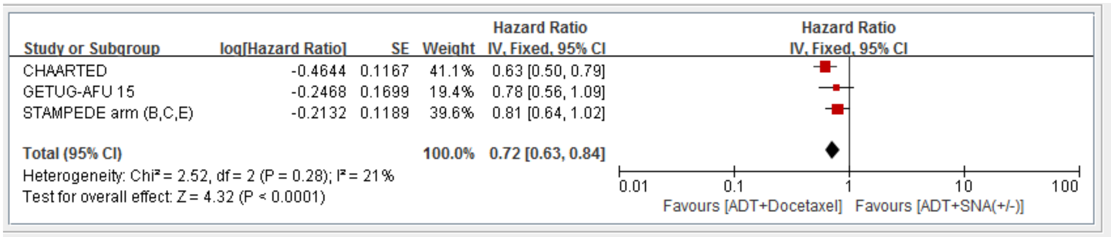

F. ADT plus docetaxel versus ADT with or without SNA on rPFS in patients with high-volume disease.

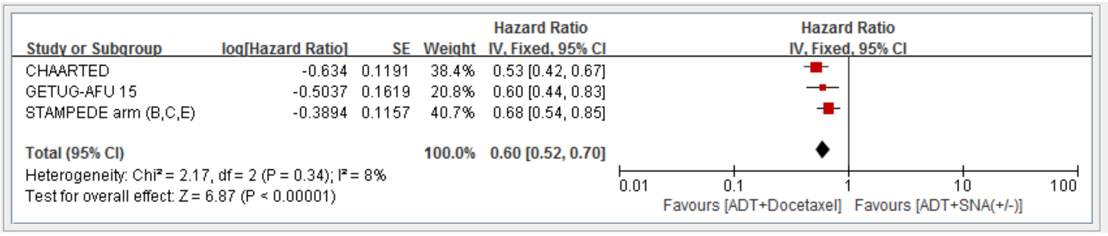

G. ADT plus abiraterone versus ADT with or without SNA on OS in patients with high-volume disease.

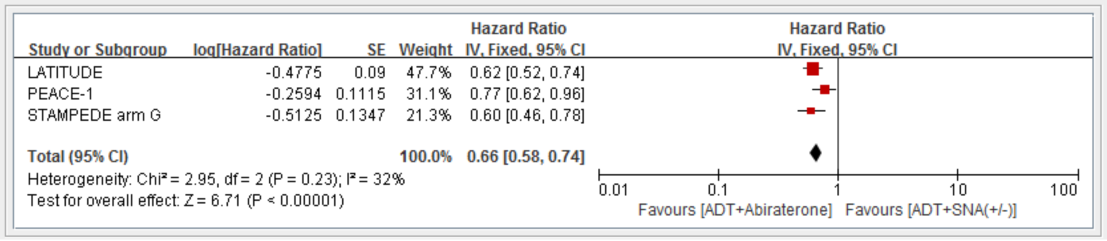

H. ADT plus abiraterone versus ADT with or without SNA on rPFS in patients with high-volume disease.

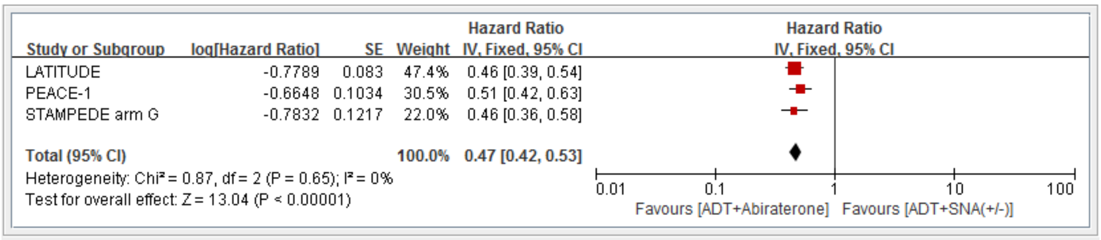

I. ADT plus enzalutamide versus ADT with or without SNA on OS in patients with high-volume disease.

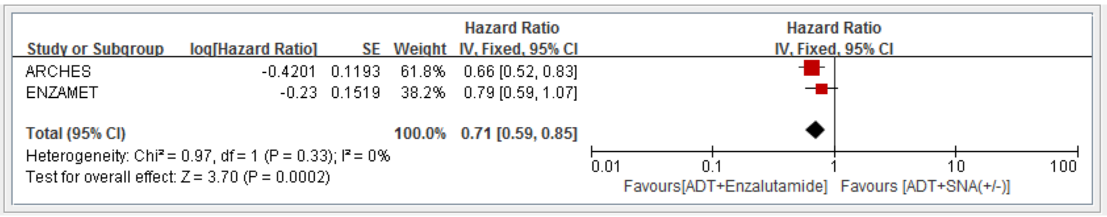

J. ADT plus enzalutamide versus ADT with or without SNA on rPFS in patients with high-volume disease.

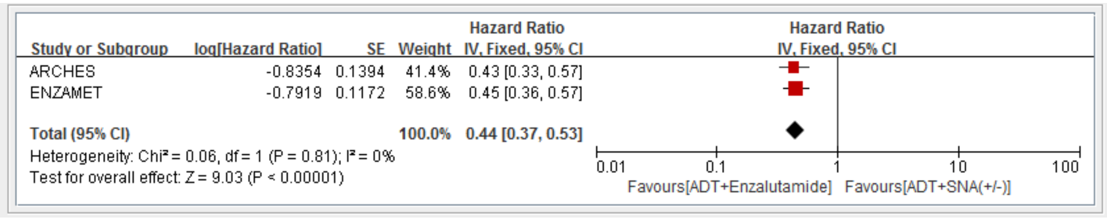

Supplementary Figure 4 Assessment of homogeneity in low-volume disease

A. ADT plus ARAT and docetaxel versus ADT plus docetaxel on OS in patients with low-volume disease.

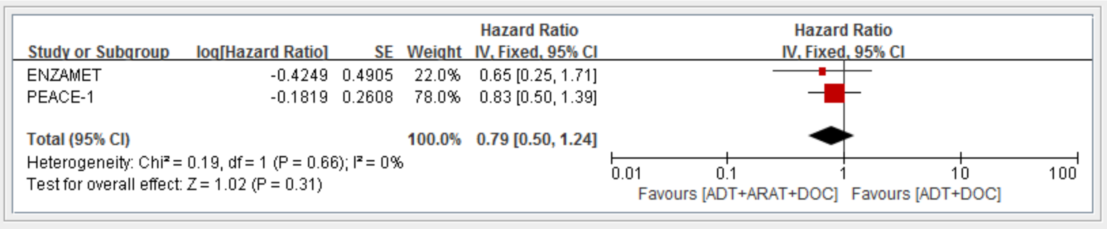

B. ADT plus ARAT and docetaxel versus ADT plus docetaxel on rPFS in patients with low-volume disease.

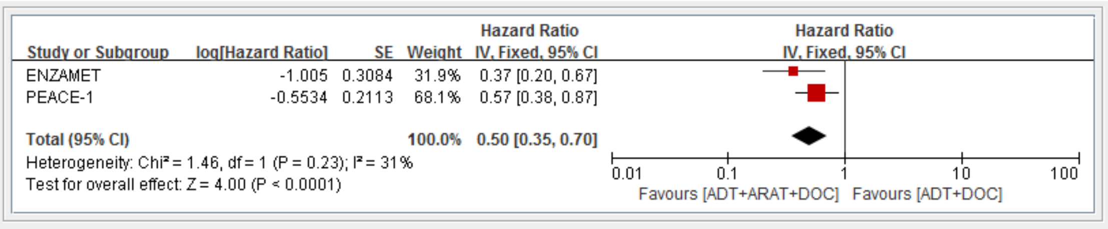

C. ADT plus ARAT versus ADT with or without SNA on OS in patients with low-volume disease.

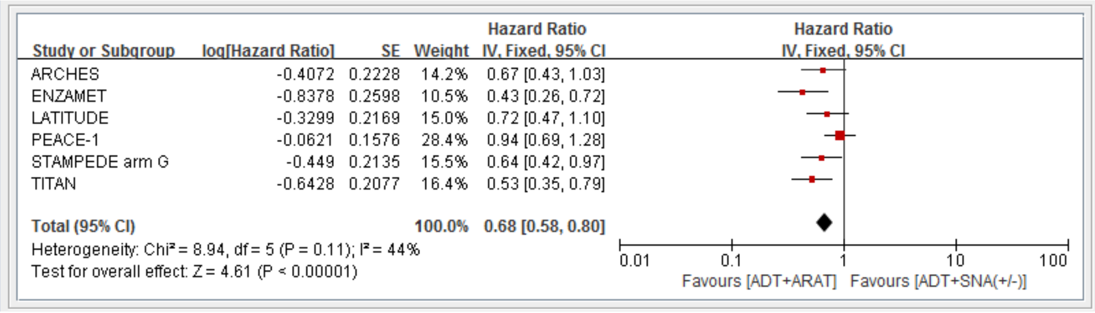

D. ADT plus ARAT versus ADT with or without SNA on rPFS in patients with low-volume disease. (ARCHES and ENZAMET trials were not included due to high heterogeneity)

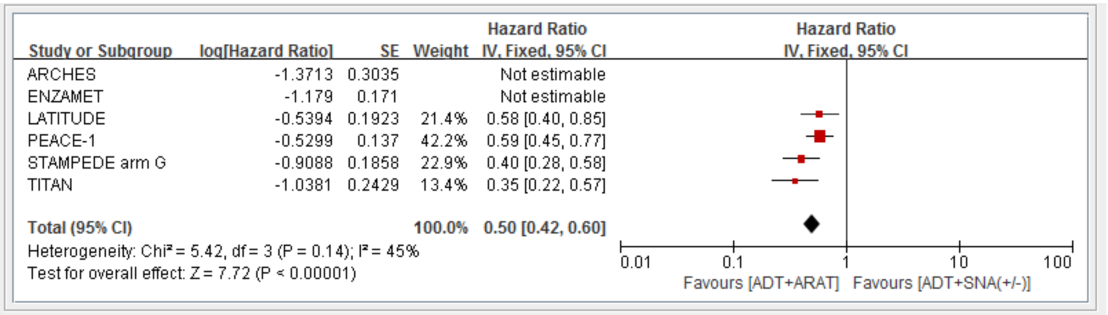

E. ADT plus docetaxel versus ADT with or without SNA on OS in patients with low-volume disease.

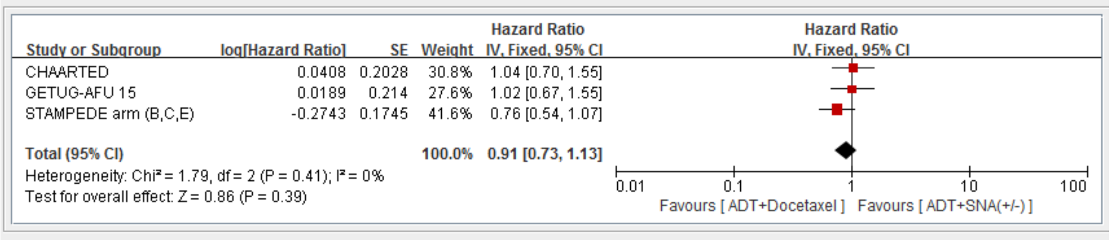

F. ADT plus docetaxel versus ADT with or without SNA on rPFS in patients with low-volume disease.

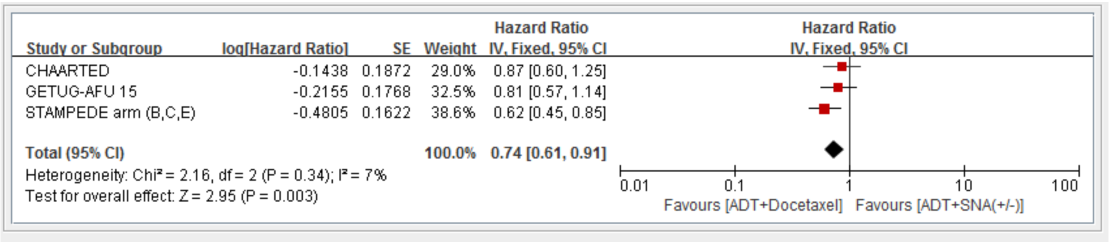

G. ADT plus abiraterone versus ADT with or without SNA on OS in patients with low-volume disease.

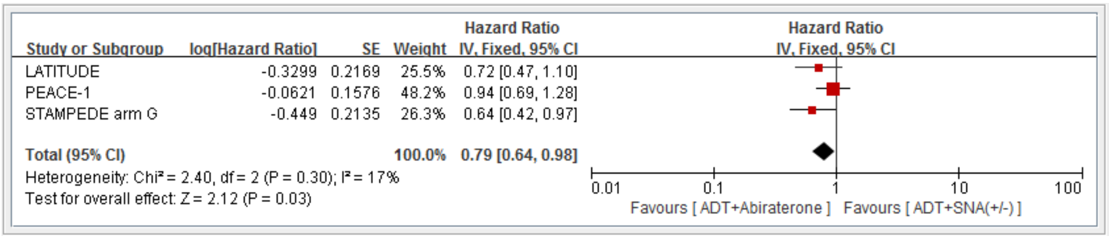

H. ADT plus abiraterone versus ADT with or without SNA on rPFS in patients with low-volume disease.

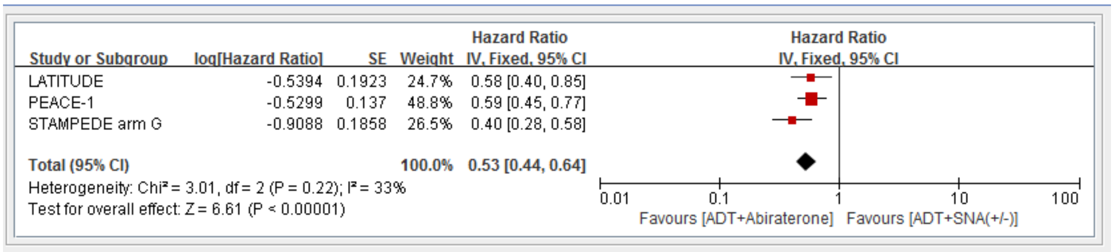

I. ADT plus enzalutamide versus ADT with or without SNA on OS in patients with low-volume disease.

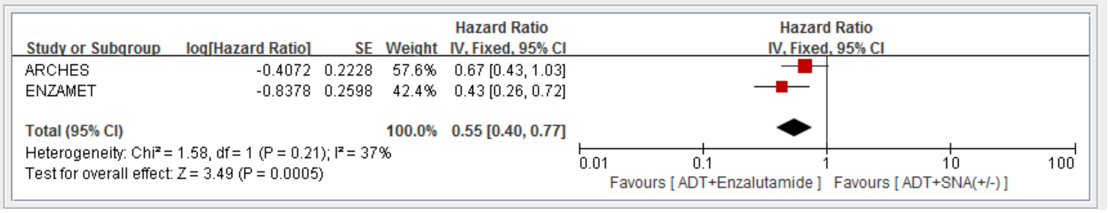

J. ADT plus enzalutamide versus ADT with or without SNA on rPFS in patients with low-volume disease.

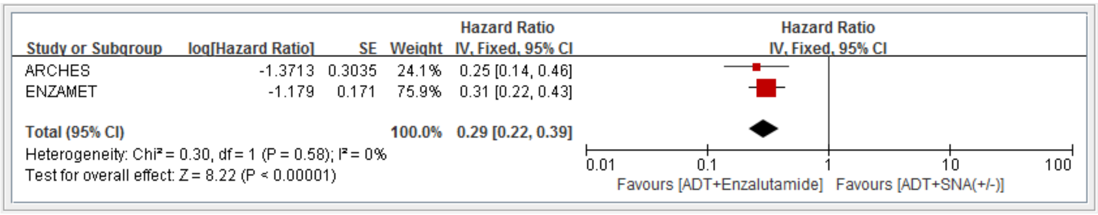

Supplementary Figure 5 Assessment of homogeneity in Adverse events

A.Combination of any AEs in ADT+Enzalutamide vs ADT+SNA(+/-) (ENZAMET trial with high heterogeneity were not included)

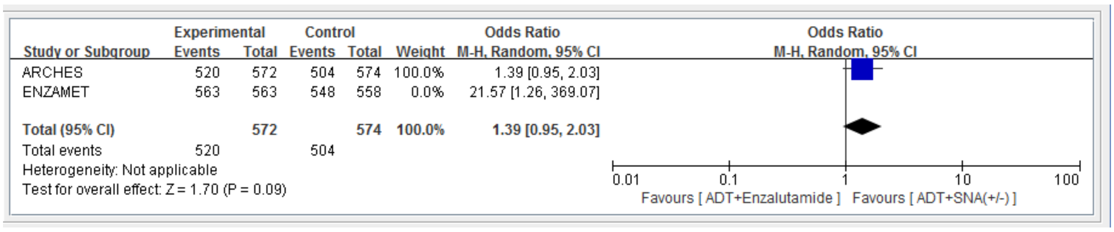

B. Combination of any AEs in ADT+Abiraterone vs ADT+SNA(+/-)

Study or Subgroup	Experimental Events	Experimental Total	Control Events	Control Total	Weight	Odds Ratio M-H, Fixed, 95% CI
LATITUDE	558	597	557	602	93.0%	1.16 [0.74, 1.80]
PEACE-1	226	226	233	237	1.3%	8.73 [0.47, 163.08]
STAMPEDE(arm G)	370	373	172	172	5.7%	0.31 [0.02, 5.97]
**Total (95% CI)**		**1196**		**1011**	**100.0%**	**1.21 [0.79, 1.84]**
Total events	1154		962			

Heterogeneity: Chi² = 2.61, df = 2 (P = 0.27); I² = 23%
Test for overall effect: Z = 0.86 (P = 0.39)

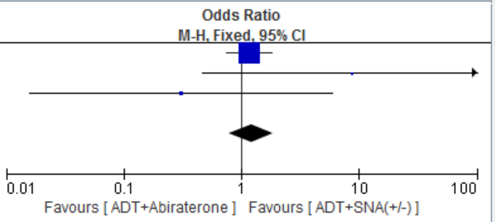

C. Combination of Grade ≥3 AEs in ADT+Enzalutamide vs ADT+SNA(+/-)

Study or Subgroup	Experimental Events	Experimental Total	Control Events	Control Total	Weight	Odds Ratio M-H, Fixed, 95% CI
ARCHES	254	572	172	574	47.8%	1.87 [1.46, 2.38]
ENZAMET	321	563	241	558	52.2%	1.74 [1.38, 2.21]
**Total (95% CI)**		**1135**		**1132**	**100.0%**	**1.80 [1.52, 2.14]**
Total events	575		413			

Heterogeneity: Chi² = 0.15, df = 1 (P = 0.70); I² = 0%
Test for overall effect: Z = 6.82 (P < 0.00001)

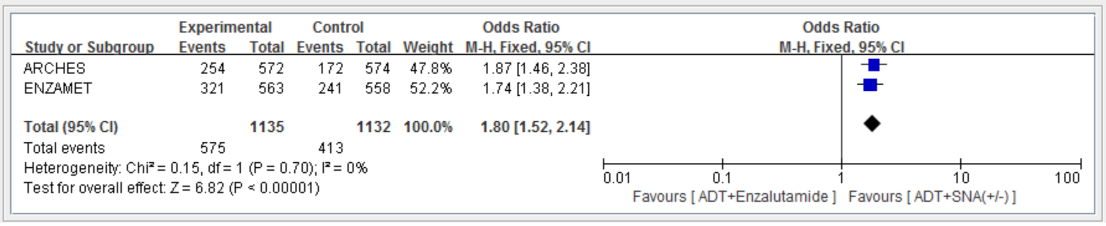

D. Combination of Grade ≥3 AEs in ADT+Abiraterone vs ADT+SNA(+/-) (PEACE-1 trial with high heterogeneity were not included)

Study or Subgroup	Experimental Events	Experimental Total	Control Events	Control Total	Weight	Odds Ratio M-H, Random, 95% CI
LATITUDE	374	597	287	602	39.1%	1.84 [1.46, 2.32]
PEACE-1	149	226	97	237	0.0%	2.79 [1.91, 4.07]
STAMPEDE(arm G)	443	948	325	960	60.9%	1.71 [1.42, 2.06]
**Total (95% CI)**		**1545**		**1562**	**100.0%**	**1.76 [1.53, 2.04]**
Total events	817		612			

Heterogeneity: Tau² = 0.00; Chi² = 0.22, df = 1 (P = 0.64); I² = 0%
Test for overall effect: Z = 7.71 (P < 0.00001)

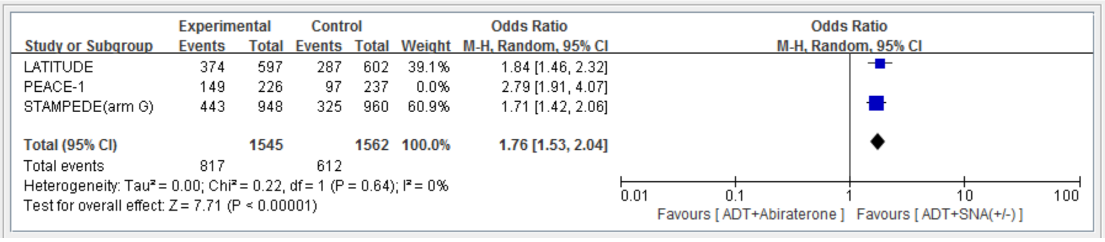

E. Combination of fatigue in ADT+Abiraterone vs ADT+SNA(+/-) (LATITUDE trial with high heterogeneity were not included)

Study or Subgroup	Experimental Events	Experimental Total	Control Events	Control Total	Weight	Odds Ratio M-H, Random, 95% CI
LATITUDE	84	592	90	602	0.0%	0.94 [0.68, 1.30]
PEACE-1	86	226	61	237	18.3%	1.77 [1.19, 2.63]
STAMPEDE(arm G)	648	948	552	960	81.7%	1.60 [1.32, 1.93]
**Total (95% CI)**		**1174**		**1197**	**100.0%**	**1.63 [1.37, 1.93]**
Total events	734		613			

Heterogeneity: Tau² = 0.00; Chi² = 0.22, df = 1 (P = 0.64); I² = 0%
Test for overall effect: Z = 5.63 (P < 0.00001)

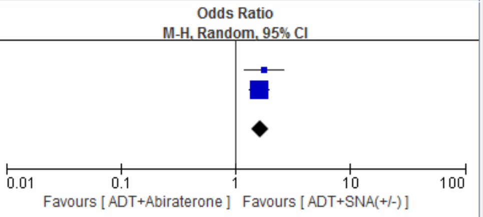

Supplementary Figure 6 Consistency analysis of direct versus indirect comparisons

A. Combination of OS in the overall population.

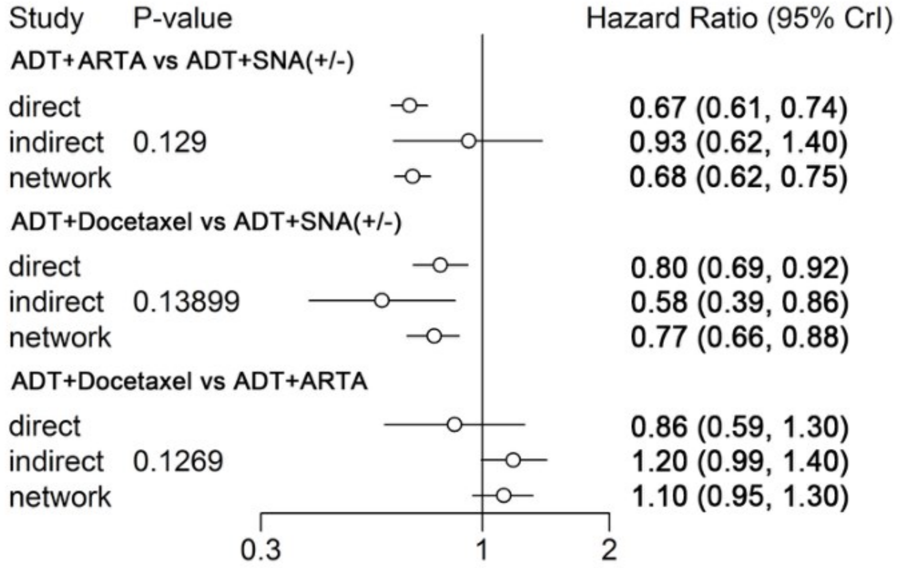

B. Combination of rPFS in the overall population.

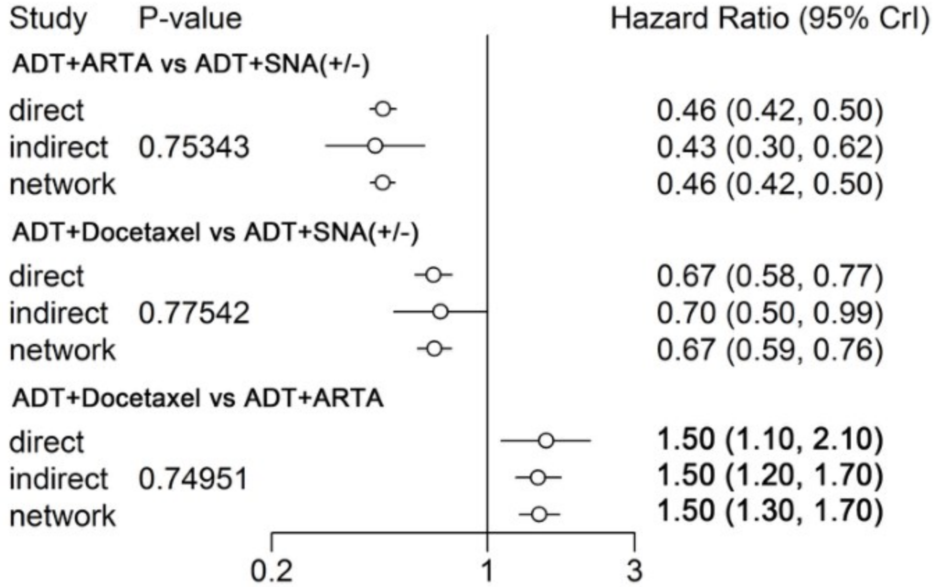

Supplementary Figure 7 Comparison of treatments in improving secondary endpoints in patients with high-volume disease.

A. Time to next skeletal-related event

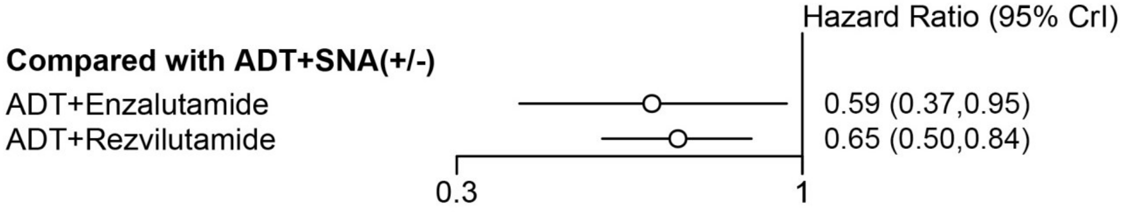

B. Time to PSA progression

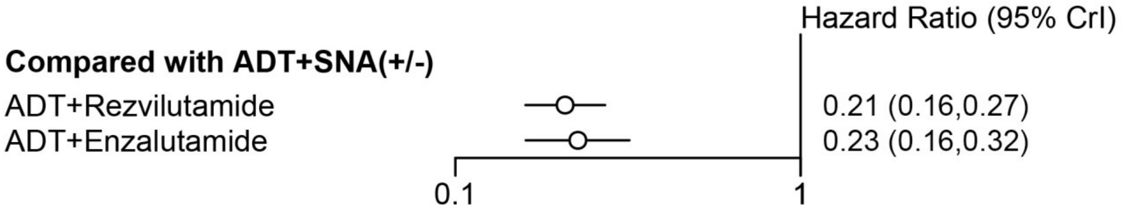

C. Time to initiation of new anti-prostate cancer therapy

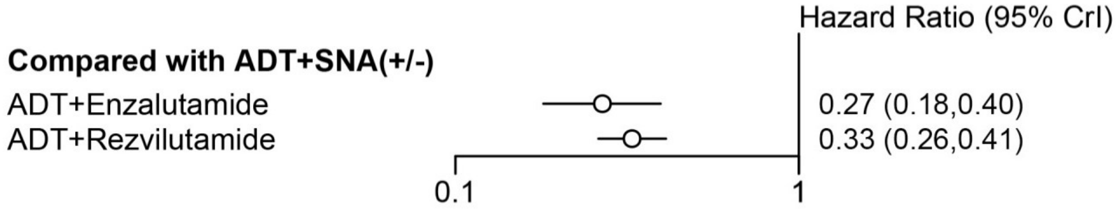

D. Time to castration resistance

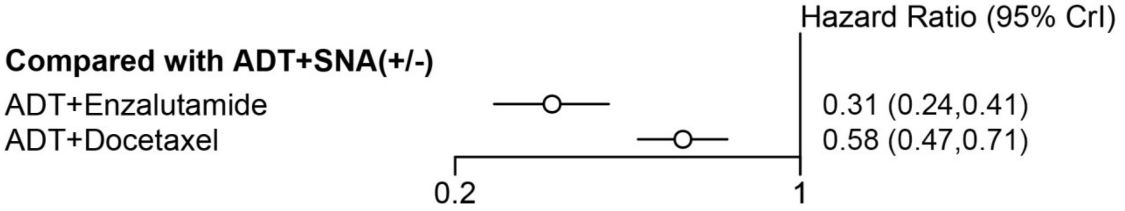

Supplementary Figure 8 Comparison of treatments in improving secondary endpoints in patients with low-volume disease.

A.Time to castration resistance

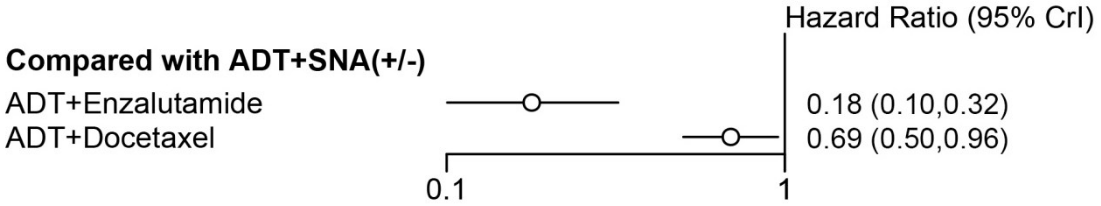

Supplementary Figure 9 Comparing treatments in improving OS in white population.
A. Network graph of trials comparison.

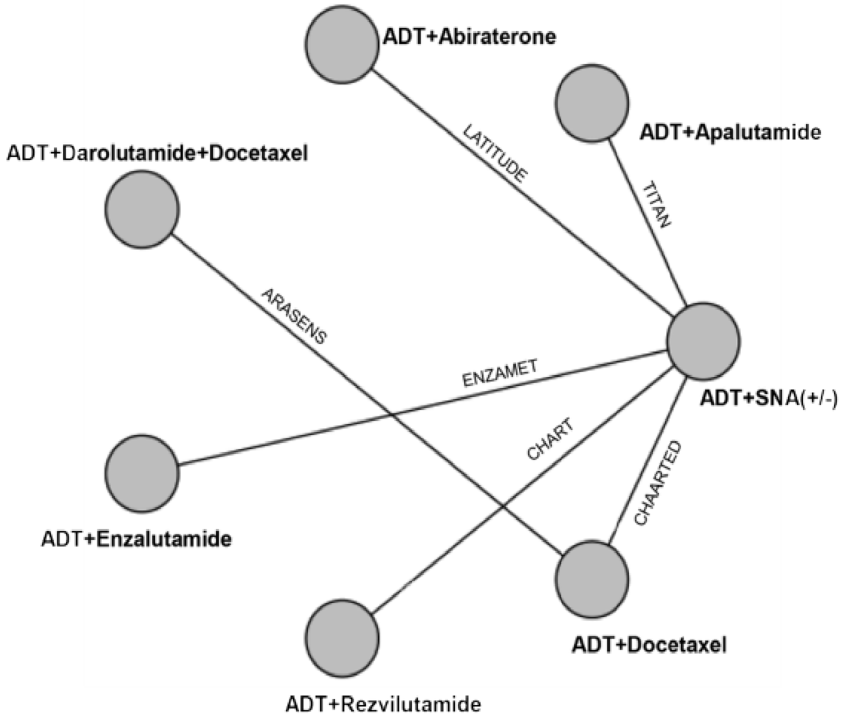

B. Forest plot representing HR for combination therapy compared with ADT with or without SNA.

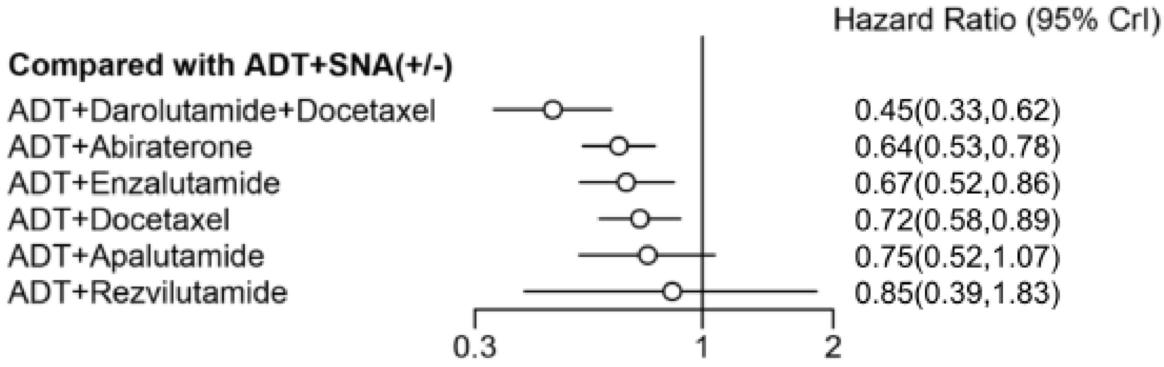

Supplementary Figure 10 Comparing treatments in improving rPFS in white population.

A. Network graph of trials comparison.

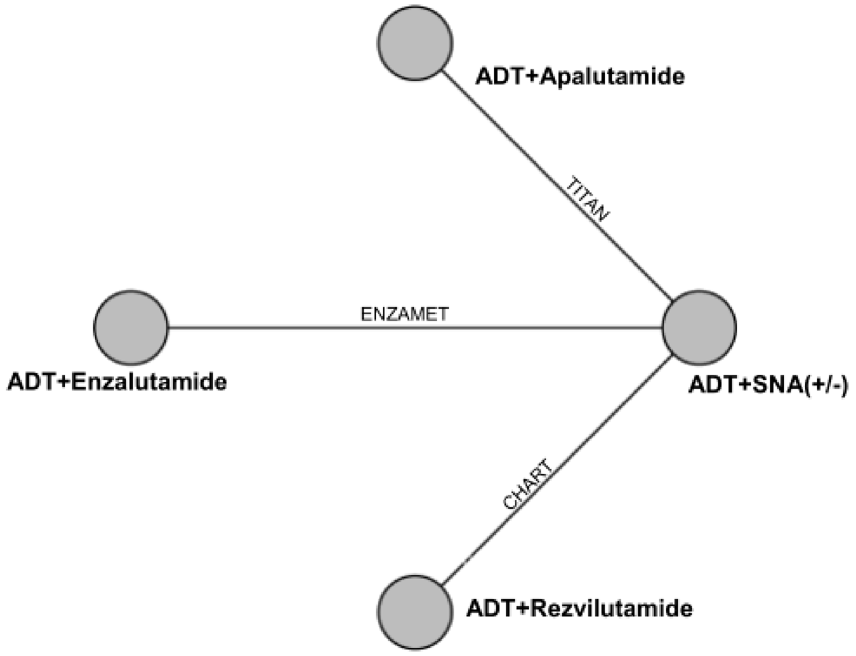

B. Forest plot representing HR for combination therapy compared with ADT with or without SNA.

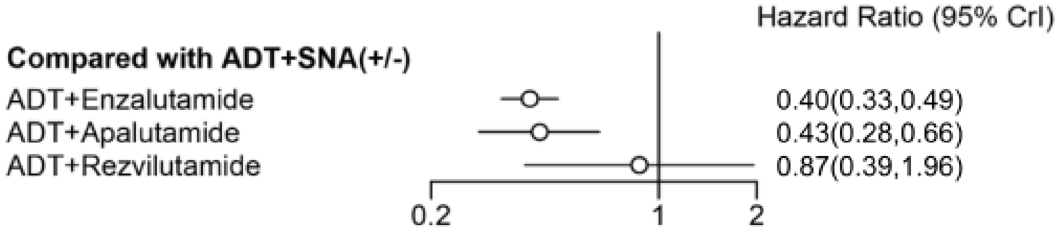

Supplementary Figure 11 Comparing treatments in improving OS in Asian population.

A. Network graph of trials comparison.

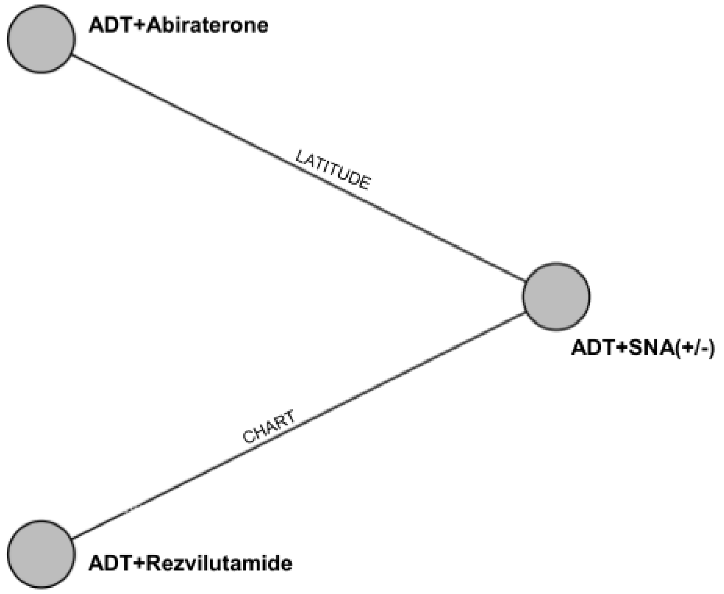

B. Forest plot representing HR for combination therapy compared with ADT with or without SNA.

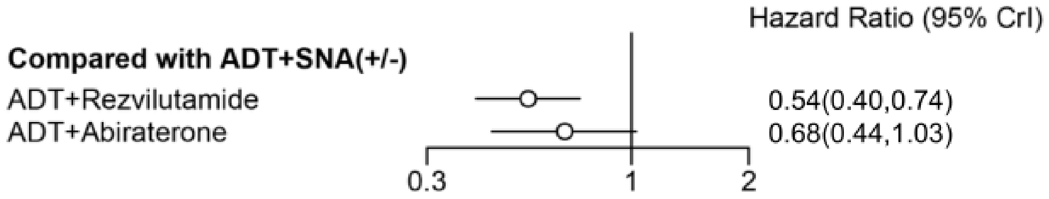

Supplement: Supplementary file 1 [file DataSheet1.PDF]
